# High-Affinity Ligands Can Trigger T Cell Receptor Signaling Without CD45 Segregation

**DOI:** 10.3389/fimmu.2018.00713

**Published:** 2018-04-09

**Authors:** Mohammad Ameen Al-Aghbar, Yeh-Shiu Chu, Bing-Mae Chen, Steve R. Roffler

**Affiliations:** ^1^Institute of Biomedical Sciences, Academia Sinica, Taipei, Taiwan; ^2^Taiwan International Graduate Program in Molecular Medicine, National Yang-Ming University, Academia Sinica, Taipei, Taiwan; ^3^Institute of Biochemistry and Molecular Biology, National Yang-Ming University, Taipei, Taiwan; ^4^Brain Research Center, National Yang-Ming University, Taipei, Taiwan; ^5^Graduate Institute of Medicine, College of Medicine, Kaohsiung Medical University, Kaohsiung, Taiwan

**Keywords:** TCR triggering, CD45 segregation, Zap70, OKT3, OKT3^MA^, scFv, TIRF microscopy, immune synapse

## Abstract

How T cell receptors (TCRs) are triggered to start signaling is still not fully understood. It has been proposed that segregation of the large membrane tyrosine phosphatase CD45 from engaged TCRs initiates signaling by favoring phosphorylation of immunoreceptor tyrosine-based activation motifs (ITAMs) in the cytoplasmic domains of CD3 molecules. However, whether CD45 segregation is important to initiate triggering is still uncertain. We examined CD45 segregation from TCRs engaged to anti-CD3 scFv with high or low affinity and with defined molecular lengths on glass-supported lipid bilayers using total internal reflection microscopy. Both short and elongated high-affinity anti-CD3 scFv effectively induced similar calcium mobilization, Zap70 phosphorylation, and cytokine secretion in Jurkat T cells but CD45 segregated from activated TCR microclusters significantly less for elongated versus short anti-CD3 ligands. In addition, at early times, triggering cells with both high and low affinity elongated anti-CD3 scFv resulted in similar degrees of CD3 co-localization with CD45, but only the high-affinity scFv induced T cell activation. The lack of correlation between CD45 segregation and early markers of T cell activation suggests that segregation of CD45 from engaged TCRs is not mandatory for initial triggering of TCR signaling by elongated high-affinity ligands.

## Introduction

T cells represent major components of the cellular arm of the adaptive immune response. Antigen presenting cells (APCs) display peptide antigens in the context of major histocompatibility complex molecules (MHC-I or MHC-II) to activate cognate T cells to develop effector functions. T cell receptor (TCR) triggering describes the initial signaling of the TCR upon binding to peptide–MHC (pMHC) complexes (1, 2).

Many models have been proposed to explain how binding of TCRs to pMHC molecules initiates signaling and activation of T cells (3), including increased proximity between CD3 immunoreceptor tyrosine-based activation motifs (ITAMs) and Src family kinases caused by surface aggregation of engaged TCRs (4) or recruitment of CD4 and CD8-associated Lck (5), induced conformational changes that expose CD3 cytoplasmic domains to phosphorylation (6, 7), while other models postulate transfer of ligated TCRs to Lck-rich lipid rafts (8). Despite numerous studies attempting to describe TCR triggering, the actual molecular mechanism remains controversial (3, 9, 10).

Kinetic segregation (KS) is a popular model of T cell activation based on the idea that large membrane receptor phosphatases, in particular CD45, are physically excluded from close contact sites of engaged TCR/pMHC complexes, thereby favoring phosphorylation of CD3 ITAMs (11, 12). Several isoforms of CD45 co-exist, each isoform has a single transmembrane domain and cytoplasmic phosphatase domains, but variably sized extracellular domains ranging from 21.6 to 55 nm (13, 14). Src family kinases as well as CD3ζ represent important substrates for CD45 dephosphorylation (15). Elongation of pMHC and membrane-tethered low-affinity anti-CD3 antibodies greatly diminishes their ability to activate T cells, consistent with enhanced dephosphorylation of CD3 ITAMs by co-localized CD45 molecules afforded by the greater intercellular distance at the engaged TCRs (16, 17). By contrast, recent reports have shown that mechanical forces specifically applied to TCRs can initiate signaling in T cells (18–20), which may not require CD45 segregation for T cell activation. In addition, we recently reported that a high-affinity membrane-tethered anti-CD3 antibody effectively triggered TCR signaling even when elongated with very long tethers (21), which is not expected to effectively cause CD45 to physically segregate from engaged TCR complexes. The role of CD45 in initiation of TCR signaling is, therefore, uncertain.

Here, we used total internal reflection fluorescence (TIRF) microscopy to investigate the dynamics of CD45 segregation from TCR microclusters. The initiation of T cell activation is induced by allowing Jurkat T cells tagged with CD3ζ GFP to interact with surfaces containing soluble anti-CD3 single-chain variable fragments (scFv) tethered on glass-supported lipid bilayer membranes. We modulated the molecular topology of proteolipid surfaces by varying the lengths of the tethered anti-CD3 scFv. By addition of a short tether corresponding to one immunoglobulin (Ig)-like domain (~3.5 nm) (22) or a longer tether derived from the CD43 extracellular domain [19–45 nm depending on the measurement method (21, 23)], we could generate different dimensions of chimeric molecules. We examined a high-affinity anti-CD3 scFv (OKT3) that can activate T cells regardless of tether dimensions and a low-affinity anti-CD3 variant (OKT3^MA^) that can activate T cells when anchored to cells *via* a short but not long tether (21). We report here that an elongated high-affinity anti-CD3 scFv induced similar calcium mobilization, IL-2 secretion and cell proliferation in Jurkat T cells as those for short anti-CD3 scFv even though it induced significantly less segregation of CD45 from engaged TCRs at early times, suggesting that CD45 segregation from engaged TCRs is not mandatory for TCR triggering.

## Materials and Methods

### Animals and Cell Lines

NOD/SCID mice were obtained from BioLASCO (Taipei, Taiwan). Animals were maintained under specific pathogen-free conditions in the Animal Core Facility of the Institute of Biomedical Sciences, Academia Sinica. 3T3 mouse fibroblasts, GP293V cells, mouse anti-human CD45 hybridoma (clone 9.4) and Jurkat T cells were from the American Type Culture Collection (Manassas, VA, USA). Jurkat T cells expressing GFP-tagged CD3ζ (24) were kindly provided by Dr. Claire Hivroz, Institute Curie, Section Recherche Pavillon Pasteur, Paris, France. All cells were cultured under aseptic conditions in media (RPMI for human primary T cells and Jurkat T cells or DMEM for other cells) (Gibco, BRL, CA, USA) supplemented with 2.98 mg/ml HEPES (USB, Cleveland, OH, USA), 2 mg/ml NaHCO_3_ (Gibco BRL, CA, USA), 100 IU penicillin, and 100 µg/ml streptomycin (Gibco, BRL, CA, USA), and 10% fetal bovine serum (FBS) (for T cells) or bovine calf serum (BCS) (for other cells) (HyClone, UT, USA).

### Antibodies

Mouse anti-human CD45 hybridoma cells were cultured in accordance with ATCC recommendations, and antibodies were collected by generation of ascites in NOD/SCID mice. Fab antibody fragments were generated by papain digestion (Pierce Fab Preparation Kit, Thermo Scientific, MA, USA). Fc fragments and undigested antibodies were removed by protein A affinity chromatography (25). Fab fragments were conjugated with DyLight650-NHS ester (Thermo Scientific, MA, USA). Rabbit anti-phospho-Zap70 antibody (clone 65E4) was from Cell Signaling (Danvers, MA, USA). Goat anti-human Ig (A + G + M), goat anti-rat IgG-FITC, and streptavidin DyLight405 were from Jackson ImmunoResearch (West Grove, PA, USA). Rat anti-HA (clone 3F10) was from (Mannheim, Germany), and biotinylated goat anti-rabbit IgG was from CHEMICON International Inc. (CA, USA). Rabbit anti-tubulin-α was from NeoMarkers, Inc. (CA, USA) and ImmunoPure^®^ goat anti-rabbit IgG-peroxidase was from Pierce Biotechnology, Inc. (IL, USA).

### Plasmids and Constructs

OKT3, OKT3^MA^, and anti-DNS scFv have been described (21, 26, 27). The scFv genes were subcloned to pLNCX retrovector (BD Biosciences, San Jose, CA, USA). An Igκ signal peptide and HA epitope tag flanked with *HindIII* and *SfiI* restriction sites were added upstream of the scFv and a 12x His tag flanked with *SalI* and *XhoI* restriction sites was cloned downstream. Then, one of the two tethers (BGP or CD43) flanked with *SalI* restriction sites were subcloned in the *SalI* site downstream of the OKT3 or OKT3^MA^ genes. Correct orientation of the tethers was confirmed by sequencing.

### Recombinant ScFv Production

Retroviruses were produced by calcium phosphate transfection of GP293V cells with retroviral vectors expressing recombinant scFv along with pVSVG (Clonetech Laboratories Inc., CA, USA) that provides the viral envelope. Packaged viruses were filtered on a 0.45-µm syringe filter and polybrene was added to a final concentration of 8 µg/ml. 3T3 cells were infected with the packaged virus, and the cells permanently expressing recombinant soluble scFv were selected in medium supplemented with 0.5 mg/ml G418 (28, 29). Stable 3T3 producer cells were cultured at confluence in medium supplemented with 0.5% BCS. Proteins in the culture medium were precipitated by addition of ammonium sulfate (Merck, Germany) to 60% of saturation and then reconstituted in binding buffer (50 mM sodium phosphate and 0.3 M NaCl, pH 7.4). Talon^®^ superflow (GE Healthcare, Sweden) was used to purify soluble scFv. Washing was done by binding buffer containing 5 mM imidazole while elution was done using 150 mM immidazole-containing buffer. Each recombinant scFv was dialyzed in three changes of PBS and then aliquots were stored at −80°C until use.

### Preparation of Lipid Small Unilamellar Vesicles (SUVs)-Ni^2+^ and ScFv-Grafted Lipid Bilayers

Small Unilamellar Vesicles containing Ni^2+^ were prepared following a previously reported method (30) using 4% 1,2-di-(9Z-octadecenoyl)-*sn*-glycero-3-[(*N*-(5-amino-1-carboxypentyl)iminodiacetic acid)succinyl] (nickel salt) (DOGS-NTA-Ni^2+^) and 96% 1,2-di-(9Z-octadecenoyl)-*sn*-glycero-3-phosphocholine (DOPC) (Avanti Polar Lipids, Inc., AL, USA). DOGS-NTA-Ni^2+^ and DOPC were mixed in chloroform and the chloroform was then evaporated in a rolling round bottom flask under strong vacuum for 3 h. The lipid film was reconstituted in Tris-saline buffer (25 mM Trsi-Cl and 150 mM NaCl, pH 8), passed 31 times through a 50 nm Whatman^®^ Nucleopore Track-Etch membrane (Sigma-Aldrich, St. Louis, MO, USA) and stored in dark tubes under N_2_ gas at 4°C and used within a week of preparation. Glass slides (#1 thickness) were washed in 70% ethanol, dried by N_2_ gas and then cleaned by oxygen plasma, 400 W, 120 mTorr for 10 min using a Nordson MARCH plasma cleaning system (CA, USA) and used within 8 h after cleaning. Proteoliposomes were prepared with 10 nM scFv in 1.6 mM SUV in Tris-saline buffer, pH 8. The proteoliposomes were kept at 4°C for at least 90 min before use. To assemble the lipid bilayers, proteoliposomes were mixed 1:1 with Tris-saline buffer containing 4 mM CaCl_2_, pH 7.0 then dropped immediately on plasma-treated glass slides assembled in the imaging chamber. After 20 min, unbound SUVs were gently washed away using TIRF imaging buffer (20 mM HEPES, 137 mM NaCl, 5 mM KCl, 0.7 mM Na_2_HPO_4_, 6 mM glucose, 2 mM MgCl_2_, and 1 mM CaCl_2_, pH 7.4) supplemented by 1% human serum albumin (Sigma-Aldrich, St. Louis, MO, USA).

### Estimating the Surface Density of ScFv on Lipid Bilayers

ScFv ligand densities on the surface of lipid bilayers were estimated based on the fluorescence intensity of stained lipid bilayer-coated glass beads that were decorated with scFv relative to Fc-specific capture antibody bead standards (Quantum^TM^ Simply Cellular^®^, Bangs Laboratories, Inc.) as described by Dustin et al. (31).

### Purifying T Cells From Human Peripheral Mononuclear Cells (PBMCs)

Human PBMCs were purified from the whole blood of healthy human donors (Taipei Blood Bank, Taiwan). RBCs were removed by ACK lysis buffer (Gibco BRL, CA), and the remaining cells were incubated in a culture dish for 30 min at 37°C to remove attached cells. Unbound cells were panned again in a culture dish precoated with goat anti-human Ig for 30 min at 37°C to remove B cells. T cells were loaded in a column packed with nylon wool fiber (Polyscience, Inc.) for 1 h at 37°C in a 5% CO_2_ humidified incubator, and T cells were collected from the column and used immediately.

### T Cell Binding Assay

Glass-bottom 96-well plates (Greiner Bio-One, Morone, NC) were filled with 1 M NaOH for 1 h, washed several times with ultra-pure distilled water and then dried by a stream of nitrogen gas. Planar lipid bilayers decorated with scFv ligands were assembled in wells. Jurkat T cells were stained with 5 µM calcein-acetoxymethyl ester (AM) (Sigma-Aldrich, St. Louis, MO, USA) for 45 min at 37°C. 1 × 10^5^ cells were added per well and incubated at 37°C for 30 min. Unbound cells were removed by serial washes with PBS. The number of bound cells was measured at four sites per well using an ImageXpress Micro XL High-Content Screening System (Molecular Devices, CA, USA). Counts from eight sites (two wells) per each group were analyzed using MetaXpress High-Content Image Acquisition & Analysis Software (Molecular Devices, CA, USA).

### Generation of Activation Beads

5 µl proteoliposomes (scFv decorated liposomes) were mixed with 10^6^ 5 µm glass beads (Bangs Laboratories, Inc., Indianapolis), and the mixture was subjected to three brief vortexes. The coated beads were washed three times using sterile PBS supplemented with 2% FBS (32). To confirm successful coating, proteoliposome-coated glass beads were stained with rabbit anti-HA IgG followed by goat anti-rabbit IgG-FITC and analyzed on a FACS Canto flow cytometer. To confirm His-Ni^2+^ binding specificity, serial concentrations of immidazole were included in washing buffer to test the potency to strip recombinant scFv from Ni^2+^-charged lipid bilayers-coated glass beads.

### T Cell Activation

Serial dilutions of glass beads coated with a lipid bilayer and decorated with scFv were mixed with 2 × 10^5^ human T cells in U-shape 96-well culture plates for 48 h in medium containing 30 ng/ml phorbol myristate acetate (PMA) (Sigma-Aldrich, St. Louis, MO, USA). After 48 h, 1 μCi ^3^H-thymidine was added per each well for 16 h before radioactivity incorporated into DNA was measured in a Topcount scintillation counter. Culture supernatants from 48 h activated T cells were collected and IL-2 concentration was measured by ELISA using a BD OptEIA kit (BD Bioscience).

### Immunoblotting

3 × 10^5^ Jurkat T cells were mixed with 1 × 10^6^ lipid-coated glass beads (5 µm) decorated with anti-CD3 scFv ligands. The activation was terminated by adding ten volumes of ice-cold PBS. Cells were pelleted immediately at 2°C, then lysed by Pierce IP lysis buffer (Thermo Fisher Scientific, MA, USA) for 1 h on ice in the presence of complete protease inhibitor cocktail (Sigma-Aldrich, St. Louis, MO, USA) and 5 mM sodium orthovanadate. Lysed cells were mixed with SDS reducing buffer and resolved in a 10% SDS-PAGE. Immunoblotting was performed on nitrocellulose membranes by rabbit anti-pZap70 and rabbit anti-tubulin-α as an internal control followed by goat anti-rabbit IgG-peroxidase at the recommended concentrations. Chemillumenscence was detected using SuperSignal^®^ West Femto Maximum Sensitivity Substrate (Thermo Scientific, MA, USA).

### TIRF Microscopy

TIRF microscopy experiments were performed using a Nikon eclipse *Ti* microscope with Perfect Focus System (PFS) coupled to a iLAS2 TIRF head with 4 wavelength lasers launch (Roper, France) or a Nicon eclipse *Ti* microscope with PFS from ANDOR Revolution WD with an Active Illumination System (ANDOR Technology Ltd., UK). 10^6^ Jurkat T cells expressing CD3ζ-GFP were stained with 1 µg mouse anti-human CD45 Fab-DyLight650 in 100 µl 2% FBS in PBS for 40 min at 4°C. Cells were washed and reconstituted in imaging buffer, warmed to physiological temperature and then dropped over functionalized lipid bilayers. Images at different positions and times were acquired using an ANDOR iXonTM Ultra 897 (ANDOR Technology Ltd., UK) or Evolve512 (Photometrics, USA) EMCCD camera, under a Nikon Apo TIRF 100× 1.49 oil objective lens. To acquire pZap70, wild-type Jurkat T cells or freshly isolated primary T cells were reconstituted in imaging buffer and then dropped over lipid bilayers on glass coverslips. After 3 min incubation at 37°C, cells were fixed for 6 min in 4% paraformaldehyde, permeabilized for 1 min by 0.1% Triton X-100 in HBSS, and then blocked by 3% BSA in PBS for 30 min. Fixed cells were stained with 5 µg/ml rabbit anti-pZap70, followed by 5 µg/ml biotinylated goat anti-rabbit IgG for 40 min at room temperature. Finally, a mixture of 5 µg/ml streptavidin-DyLight405 and 5 µg/ml mouse anti-human CD45 Fab-DyLight650 was added to the fixed cells for 40 min at room temperature. All washes and dilutions were performed with 3% BSA in PBS. Image acquiring was done using the same TIRF imaging system.

### Calcium Flux Detection

Ca^2+^ flux was measured under 20× magnification with a Leica microscope and accompanying Metafluor software (Molecular Devices, CA, USA). Briefly, cells were stained with 1 µM Fura-2 AM (Invitrogen, CA, USA) in the presence of 5 µM Pluronic F-127 (Sigma-Aldrich, St. Louis, MO, USA) in HBSS (Gibco, BRL, CA, USA) for 30 min at room temperature. Cells were washed three times with HBSS and kept for an additional 30 min at room temperature before performing the experiment. Cells were dropped on the assembled lipid bilayers covered with TIRF imaging buffer supplemented with 30 ng/ml PMA, and the ratiometric images were acquired every 15 s for 20 min. The specificity of the calcium response was ensured by pretreating Jurkat T cells with 25 µM PP2 (Abcam, Cambridge, MA, USA), a potent inhibitor of p56lck tyrosine phosphorylation, for 30 min at 37°C before dropping the cells. The drug concentration was maintained in the imaging buffer during the experiment.

### Data Analysis

All TIRF and immunofluorescence images were processed using Metamorph or Metafluor software (Molecular Devices, CA, USA). TCR or pZap70 and CD45 co-localization at different times was examined at both the whole cell and the microcluster level. The integrated intensity threshold was determined for the GFP or DyLight405 channel as well as the DyLight650 channel, then the intensity measurement co-localization function from Metamorph was used to determine the overlapping between channels, considering DyLight650 channel as 100%. To measure co-localization percentage between microclusters, the threshold was determined at the GFP or DyLight405 channel, then regions were drawn around each microcluster. For technical limitations, all regions smaller than 0.03 µm^2^ were excluded. Then using JACoP plugin in ImageJ software (NIH), co-localization was determined between overlapping pixels by calculating the M1 Mander’s coefficient. All the co-localization data were plotted as percentage, either at cell or at microcluster levels.

Statistical analysis was assessed with GraphPad Prism 5 software. Significance of differences between mean values was estimated using one-way ANOVA (Kruskal–Wallis one-way analysis of variance), or by non-parametric unpaired *t*-test with Welch’s correction. The statistical significance was set at *p* < 0.05.

## Results

### Production of Soluble Anti-CD3ε ScFv Ligands

**Figure 1:**
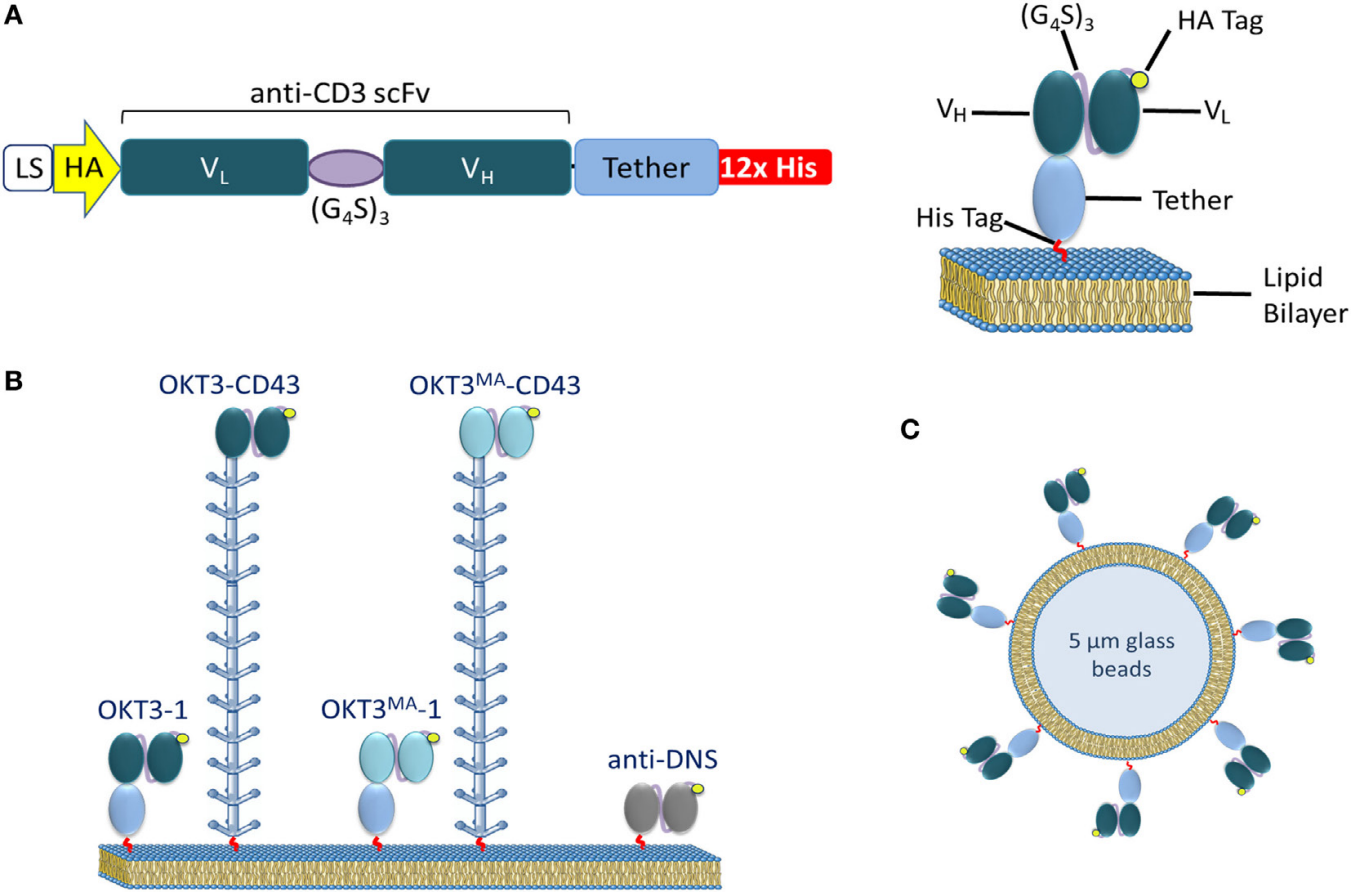
Lipid membrane-tethered anti-CD3 antibodies. **(A)** Left: a retroviral cassette expressing soluble anti-CD3 scFv. The cassette includes an IgGκ leader sequence, a sequence coding for an HA tag, the anti-CD3 scFv V_L_ and V_H_ regions connected by a GS linker (G_4_S)_3_, followed by a short or long tether and 12× His tag. Right: a schematic drawing of the scFv attached to a lipid bilayer by Ni-His affinity interactions. **(B)** A schematic drawing of the ligands used: OKT3 or OKT3^MA^ scFv elongated by an immunoglobulin-like domain (BGP) or the CD43 ectodomain. **(C)** A schematic drawing of artificial APCs formed from 5 µm glass beads coated with a lipid bilayer decorated with anti-CD3 scFv.

We prepared soluble anti-CD3 scFv antibodies with either a short (BGP) or long (CD43) tether (Figure 1A). A twelve residue C-terminal histidine tag was used to anchor the antibodies on NTA-lipid supported bilayers to vary the spatial distance between the lipid bilayer and T cell interface. OKT3 is a high-affinity anti-CD3 antibody whereas OKT3^MA^, which possesses two amino acid substitutions in the heavy chain variable region complementary determining regions, displays about 250-fold lower binding affinity as compared to OKT3 (21) (Figure 1B). This triggering system was applied for real-time TIRF imaging to track the fate of CD45 upon anti-CD3/TCR ligation. A soluble control scFv with a C-terminal histidine tag was also prepared from an antibody that does not bind T cells (anti-DNS). In addition to the planar lipid bilayers, we prepared artificial APCs using 5 µm glass beads coated with lipid bilayers decorated with the soluble scFv ligands (Figure 1C). These beads were used to measure scFv density and T cell activation.

### Ligand Elongation on Lipid Bilayers Has No Effect on Jurkat T Cell Binding

We confirmed the presence of anti-CD3 scFv on lipid-coated glass beads by detecting the N-terminal HA tag on the scFv. Comparable densities of anti-CD3 scFv ligands were conjugated to the lipid bilayers as well (Figure S1A in Supplementary Material). The scFv ligands could be competitively stripped from the Ni^2+^ functionalized lipid on the glass beads by imidazole, demonstrating that the scFv were bound *via* the C-terminal polyhistidine tag (Figure S1B in Supplementary Material).

**Figure 2:**
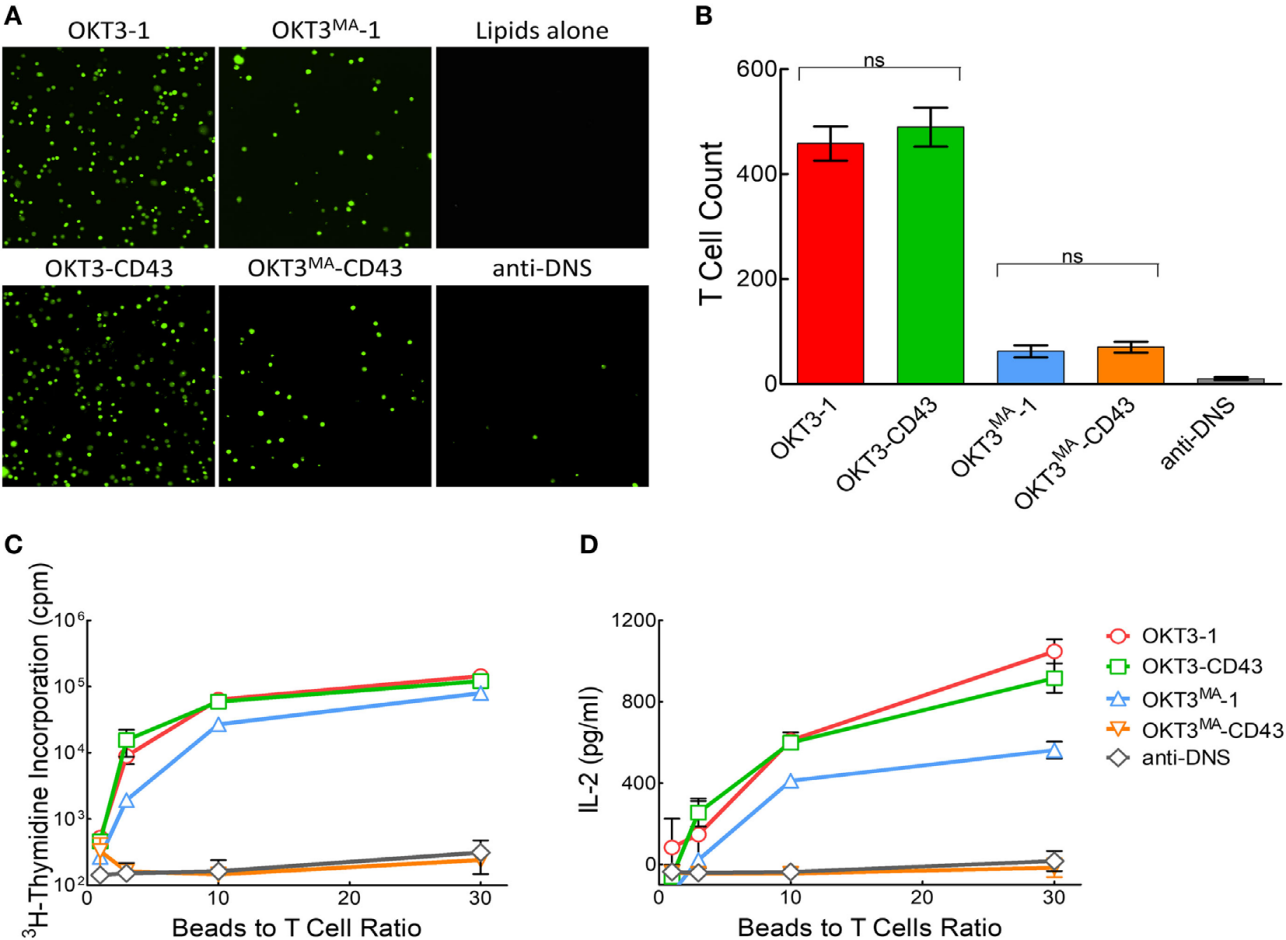
T cell activation by anti-CD3 scFv tethered to lipid bilayer-coated glass beads. **(A)** Fluorescent imaging of calcein-labeled Jurkat T cells binding to anti-CD3 scFv or control anti-DNS scFv tethered to planar lipid bilayer-coated glass surfaces. **(B)** Mean number of bound cells (*n* = 8). Bars, SD. ns, not significantly different. **(C)** 2 × 10^5^ primary human T cells were incubated with the indicated ratios of lipid bilayer-coated glass beads decorated with anti-CD3 scFv. Results show T cell proliferation as measured by ^3^H-thymidine incorporation (*n* = 4). Bars, SD. **(D)** IL-2 concentration after 48 h in wells containing 2 × 10^5^ primary human T cells incubated with the indicated ratios of lipid bilayer-coated glass beads decorated with anti-CD3 scFv (*n* = 3). Bars, SD.

The binding of calcein-labeled Jurkat T cells to glass-supported lipid bilayers decorated with the same density of short or long anti-CD3 scFv was examined at 37°C. After washing unbound T cells, the remaining number of bound T cells was counted at eight different positions for each ligand (Figure 2A). More Jurkat T cells bound to bilayers coated with high-affinity ligands (OKT3-1 and OKT3-CD43) as compared to bilayers coated with low-affinity scFv (OKT3^MA^-1 and OKT3^MA^-CD43), consistent with the binding affinities of the scFv (Figure 2A). However, there was no statistical difference in the binding of T cells to short or long scFv with the same affinities (Figure 2B), indicating that T cells binding is affected by the ligand affinity, but not the ligand dimensions.

### The Affinity and Topology of Lipid-Tethered Anti-CD3 ScFv Determines T Cell Activation

We previously reported that activation of naïve T cells by 3T3 fibroblasts expressing membrane-tethered anti-CD3 scFv displayed strong dependence on tether dimensions for low but not high-affinity anti-CD3 scFv (21). We, therefore, examined if a similar trend was observed for anti-CD3 antibodies tethered to a lipid bilayer. The high-affinity OKT3 and low-affinity OKT3^MA^ scFv were tethered to lipid bilayer-coated glass beads and used to activate human T cells isolated from PBMCs. Similar to the results observed in our previous study (21), both high- and low-affinity short ligands (OKT3-1 and OKT3^MA^-1) and the high-affinity elongated scFv (OKT3-CD43) induced T cell proliferation (Figure 2C) and IL-2 secretion (Figure 2D) whereas the elongated low-affinity scFv (OKT3^MA^-CD43) did not activate T cells.

**Figure 3:**
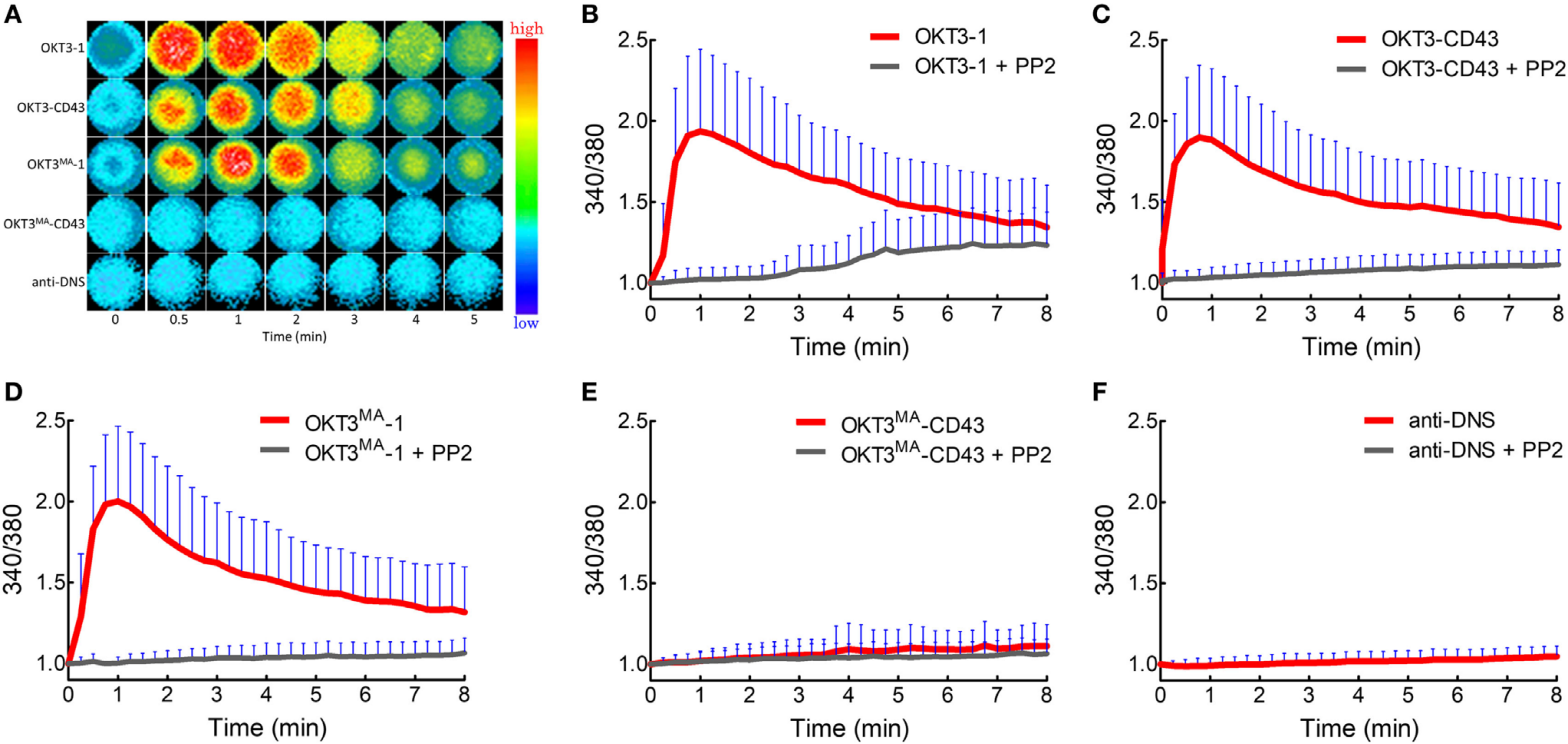
Calcium flux in Jurkat T cells stimulated with membrane-tethered anti-CD3 scFv. Jurkat T cells were dropped on planar lipid bilayers decorated with anti-CD3 scFv in the presence or absence of Lck inhibitor (PP2). The emission was measured from Fura-2AM labeled Jurkat T cells as the ratio between 340 and 380 nm at different times (min). **(A)** Ca^2+^ flux measured from individual Fura-2AM stained Jurkat T cells. A heat map for Ca^2+^ from individual Jurkat T cells over time. Mean ratiometric fluorescence of Fura-2AM loaded Jurkat T cells triggered by membrane-tethered OKT3-1 **(B)**, OKT3-CD43 **(C)**, OKT3^MA^-1 **(D)**, OKT3^MA^-CD43 **(E)**, or anti-DNS control scFv **(F)** (*n* = 52 and 30 cells without PP2 and with PP2 inhibition, respectively). Bars, SD.

Calcium flux is one of the earliest indicators of TCR triggering (33). pZap70 drives the cascade signaling that leads to *phosphatidylinositol-4, 5 bisphosphate (*PIP2) hydrolysis to *inositol 1, 4, 5-triphosphate (*IP3), which binds to endoplasmic reticulum Ca^2+^-sensitive channels, inducing Ca^2+^ flux to the cytoplasm in a matter of seconds (34). We, therefore, examined calcium mobilization in Jurkat T cells stimulated by short or long OKT3 and OKT3^MA^ scFv on glass-supported lipid bilayers. Cells were first labeled with the calcium-sensitive dye Fura-2AM and then dropped over lipid bilayers coated with the anti-CD3 scFv. Measurement of calcium mobilization in individual T cells demonstrated strong triggering of calcium mobilization by both short and long high-affinity OKT3 scFv as well as by the short low-affinity OKT3^MA^ scFv but lack of calcium mobilization by the elongated OKT3^MA^-CD43 (Figure 3A). Examination of a larger number of individual T cells also demonstrated that OKT3 scFv with both short (Figure 3B) and long (Figure 3C) tethers stimulated prolonged Ca^2+^ mobilization in Jurkat T cells upon contacting planar lipid bilayers. By contrast, OKT3^MA^-1 (Figure 3D) but not OKT3^MA^-CD43 (Figure 3E) induced robust release of calcium in Jurkat T cells. As expected, lipid bilayers decorated with the non-binding control anti-DNS scFv were unable to induce detectable amounts of Ca^2+^ mobilization in Jurkat T cells (Figure 3F). Addition of the Lck inhibitor PP2 blocked calcium mobilization in all cases, consistent with triggering via the TCR complex. Taken together, these results show that the high-affinity OKT3 scFv triggered T cell signaling regardless of tether dimensions while the low-affinity OKT3^MA^ scFv only triggered TCR signaling when tethered to lipid bilayers *via* the short tether.

### TCR Triggering Is Initiated by an Elongated High-Affinity Ligand Without Clear CD45 Segregation

**Figure 4:**
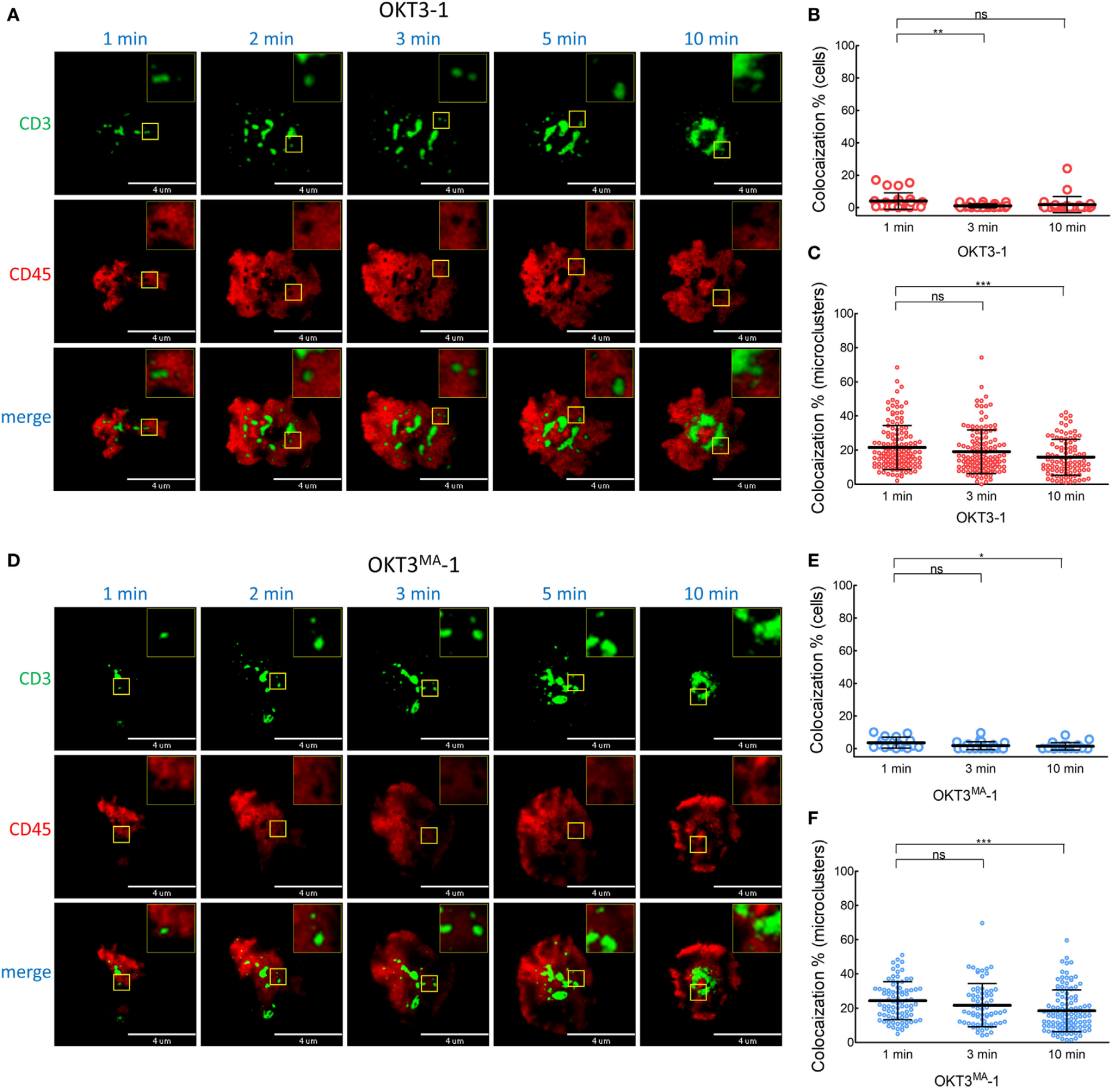
CD45 is rapidly segregated from T cell receptor complexes when T cells are activated with short ligands. **(A)** A representative cell was imaged for CD3 (green) and CD45 (red) by TIRF microscopy at 1, 2, 3, 5, and 10 min after contact with OKT3-1 on a planar lipid bilayer. **(B)** Co-localization percentage between CD3 and CD45 measured by intensity of overlapped pixels for whole Jurkat cell activated by OKT3-1 (*n* = 26 cells, representative of three independent experiments). **(C)** Co-localization percentage between CD3 and CD45 measured by intensity of the overlapped pixels for individual microclusters obtained from Jurkat cells activated by OKT3-1 (*n* = 26 cells, representative of three independent experiments). **(D)** A representative cell was imaged for CD3 (green) and CD45 (red) by TIRF microscopy at 1, 2, 3, 5, and 10 min after contact with OKT3^MA^-BGP on a planar lipid bilayer. **(E)** Co-localization percentage between CD3 and CD45 measured by intensity of overlapped pixels for whole Jurkat cell activated by OKT3^MA^-BGP (*n* = 22 cells, representative of three independent experiments). **(F)** Co-localization percentage between CD3 and CD45 measured by intensity of the overlapped pixels for individual microclusters obtained from Jurkat cells activated by OKT3^MA^-BGP (*n* = 22 cells, representative of three independent experiments). Bars, SD.

We expect that there should be differential segregation of CD45 for activating (OKT3-1, OKT3-CD43, and OKT3^MA^-1) versus non-activating (OKT3^MA^-CD43) anti-CD3 scFv if CD45 segregation is required for T cell activation. We, therefore, used live-cell TIRF imaging of Jurkat T cells on glass-supported lipid bilayers that were grafted with different anti-CD3 scFvs. CD45 rapidly (within seconds) segregated from TCR microclusters in Jurkat T cells that were incubated with short OKT3-1 (Figure 4A; Video S1 in Supplementary Material) and OKT3^MA^-1 (Figure 4D) scFv. Minimal co-localization of CD3 and CD45 was observed on the surface of T cells from 1 to 10 min (Figures 4B,E). Measurement of CD45 in individual TCR clusters also revealed little co-localization (Figures 4C,F).

**Figure 5:**
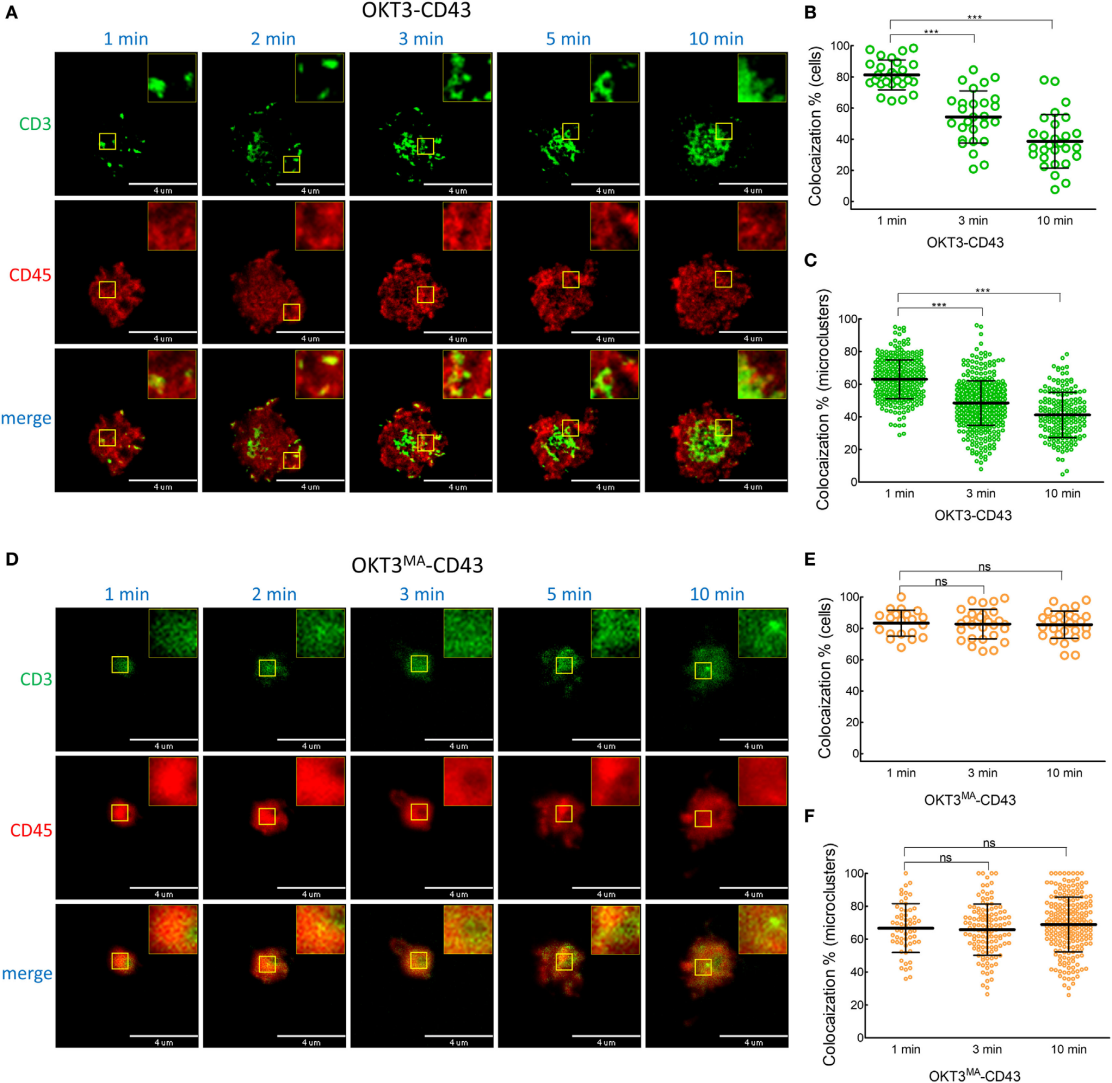
Co-localized CD45 and CD3 by elongated ligands start segregation only by productive activation. **(A)** A representative cell was imaged for CD3 (green) and CD45 (red) by TIRF microscopy at 1, 2, 3, 5, and 10 min after contact with OKT3-CD43 on a planar lipid bilayer. **(B)** Co-localization percentage between CD3 and CD45 measured by intensity of overlapped pixels for whole Jurkat cell activated by OKT3-CD43 (*n* = 27 cells, representative of three independent experiments). **(C)** Co-localization percentage between CD3 and CD45 measured by intensity of the overlapped pixels for individual microclusters obtained from Jurkat cells activated by OKT3-CD43 (*n* = 27 cells, representative of three independent experiments). **(D)** A representative cell was imaged for CD3 (green) and CD45 (red) by TIRF microscopy at 1, 2, 3, 5, and 10 min after contact with OKT3^MA^-CD43 on a planar lipid bilayer. **(E)** Co-localization percentage between CD3 and CD45 measured by intensity of overlapped pixels for whole Jurkat cell activated by OKT3^MA^-CD43 (*n* = 20, 29, and 30 cells for the three time points, representative of three independent experiments). **(F)** Co-localization percentage between CD3 and CD45 measured by intensity of the overlapped pixels for individual microclusters obtained from Jurkat cells activated by OKT3^MA^-CD43 (*n* = 17 cells, representative of three independent experiments).

**Figure 6:**
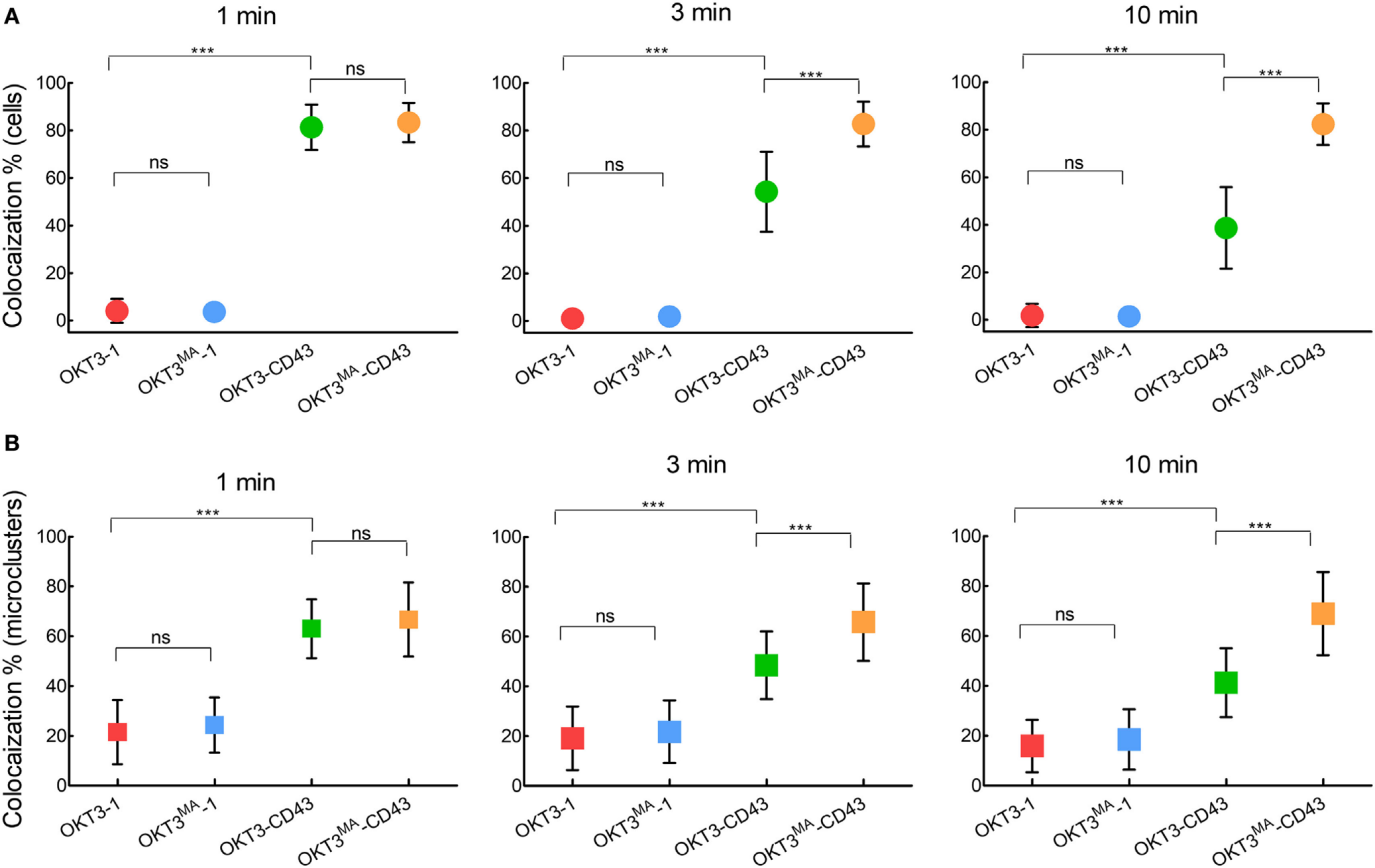
CD45 segregation from CD3 occurs after T cell activation by elongated ligands and increases with time. We compared segregation of CD3 and CD45 induced by ligands with the same length and different affinities at 1, 3, and 10 min as measured by **(A)** Co-localization percentage between CD3 and CD45 measured by intensity of overlapped pixels for individual Jurkat T cells. **(B)** Co-localization percentage between CD3 and CD45 measured by intensity of the overlapped pixels for individual microclusters obtained from Jurkat cells (*n* = 26, 22, 27 and 20 cells for OKT3-BGP, OKT3^MA^-BGP, OKT3-CD43, and OKT3^MA^-CD43, respectively). Bars, SD.

When Jurkat T cells were incubated for 1 min on lipid bilayers decorated with the long high-affinity ligand (OKT3-CD43), CD45 mostly co-localized with TCR microclusters (Figures 5A–C; Video S2 in Supplementary Material). CD45 also largely co-localized with TCR microclusters in Jurkat cells incubated for 1 min on lipid bilayers decorated with the low affinity elongated ligand (OKT3^MA^-CD43), but with obviously lower spreading of T cells (Figures 5D–F). CD45 partially segregated from TCR microclusters when Jurkat T cells contacted the high-affinity elongated ligand OKT3-CD43 for 3–10 min, suggesting that CD45 segregation follows rather than initiates TCR triggering (Figures 5B,C; Figure S2 in Supplementary Material). By contrast, the elongated low-affinity ligand OKT3^MA^-CD43, which cannot activate T cells, showed a constant pattern of co-localization for up to 10 min (Figures 5E,F). Comparison of the co-localization index at 1, 3, and 10 min revealed that there were no significant differences between OKT-1 and OKT^MA^-1 at any time; both short ligands induced strong segregation of CD45 from TCR clusters at all times (Figure 6). Likewise, there was no significant difference in CD45 segregation induced by OKT-1 and OKT^MA^-1 at 1 min; CD45 mostly co-localized with TCR clusters for both elongated anti-CD3 scFv. However, OKT3-CD43 induced significantly more segregation of CD45 as compared to OKT3^MA^-CD43 at 3 and 10 min (Figure 6). Of note, OKT3-CD43 induced significantly less segregation of CD45 from TCR microclusters as compared to OKT3-1 at 1, 3, and 10 min.

### CD45 Segregation from Phosphorylated Zap70

**Figure 7:**
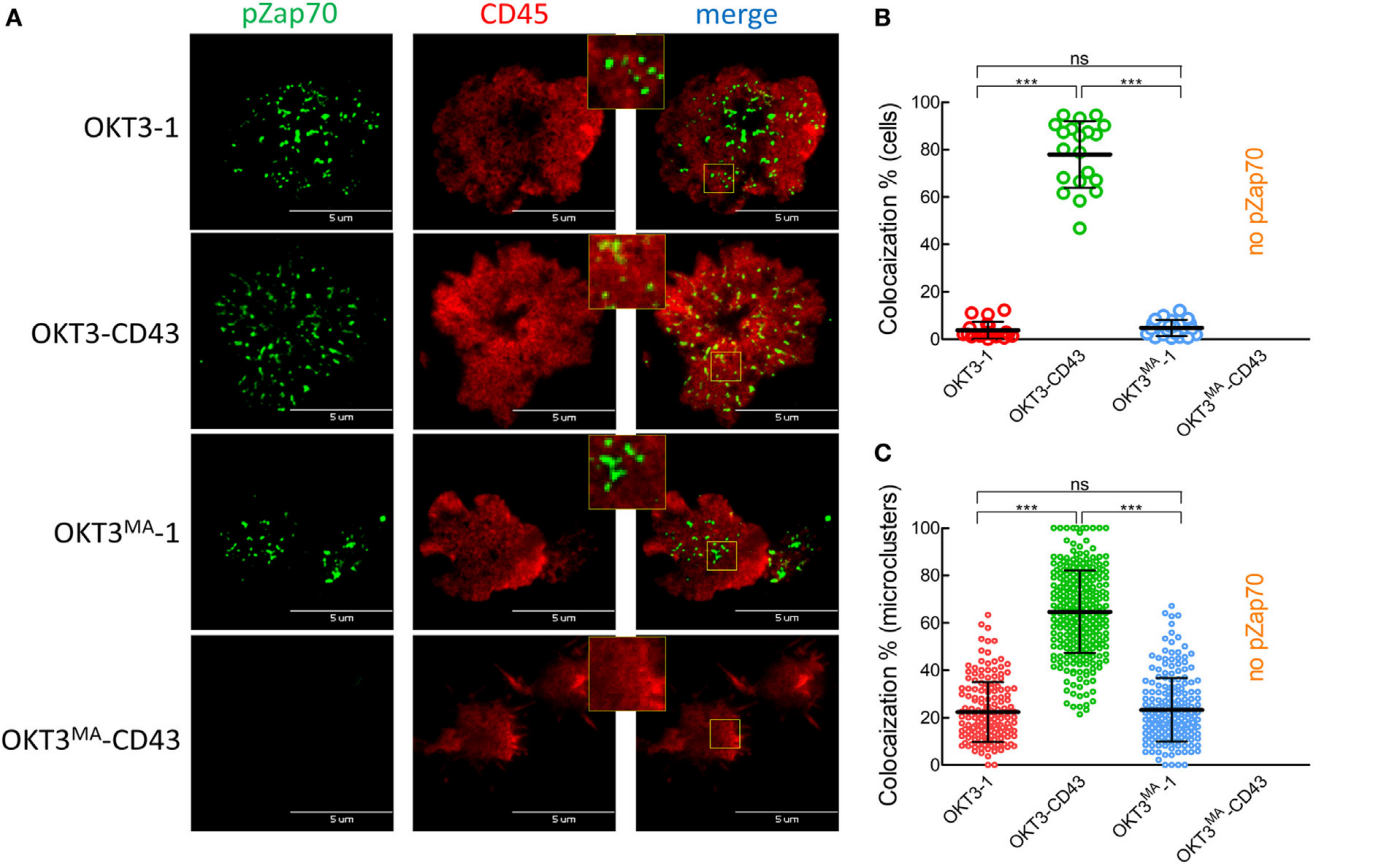
CD45 partially segregates from pZap70 when triggering T cells with OKT3-CD43. **(A)** Jurkat T cells were placed over planar lipid bilayers decorated with the scFv ligands, fixed after 3 min, and stained for pZap70 (green), and CD45 (red). Small yellow squares are enlarged. **(B)** Co-localization percentage between pZap70 and CD45 measured by intensity of overlapped pixels for whole Jurkat cells activated by different ligands. **(C)** Co-localization percentage between pZap70 and CD45 measured by intensity of the overlapped pixels for individual microclusters obtained from Jurkat cells and activated by different ligands (*n* = 20 cells per ligand). Bars, SD.

We further examined the relationship between CD45 segregation and Zap70 phosphorylation as a marker of activated TCRs (35). Consistent with the T cell proliferation, cytokine secretion and calcium mobilization results, evident pZap70 was generated in T cells incubated on lipid bilayers decorated with OKT3-1, OKT3^MA^-1, and OKT3-CD43, while there was no detectable pZap70 when Jurkat T cells were incubated on lipid bilayers decorated with OKT3^MA^-CD43 (Figure 7A). CD45 clearly segregated from pZap70 in T cells incubated on lipid bilayers containing the short OKT3-1 and OKT3^MA^-1 scFv but segregated significantly less in T cells incubated on lipid bilayers with elongated OKT3-CD43 scFv (Figures 7B,C), consistent with a lack of strong association between CD45 segregation and TCR triggering.

### Low Ligand Density Showed Similar Patterns of Activation and CD45 Segregation

**Figure 8:**
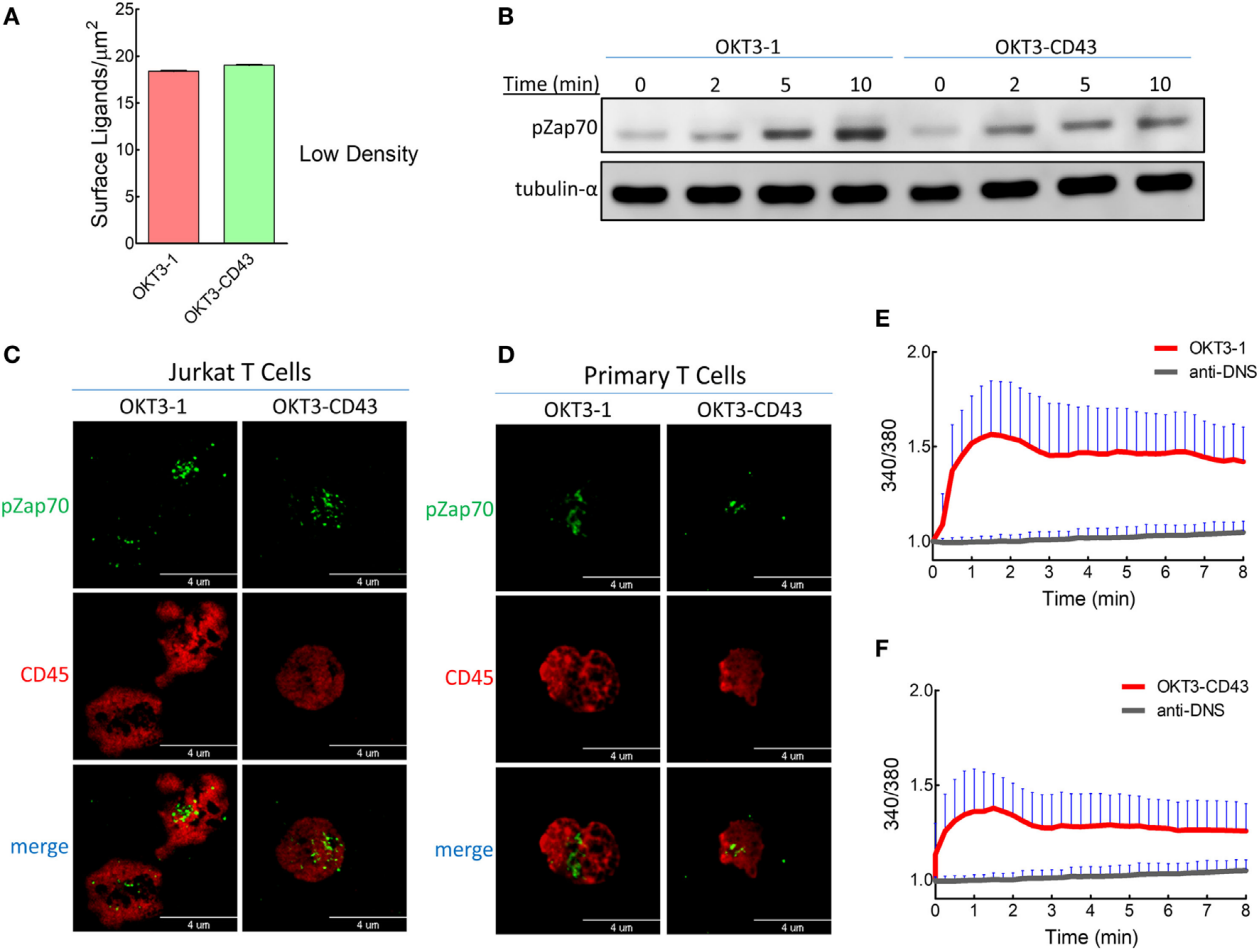
T cell receptors can be triggered by low densities of high-affinity ligands. **(A)** The estimated densities of anti-CD3 scFv were measured by comparing fluorescent intensities of anti-CD3 scFv on lipid bilayer-coated glass beads relative to standard beads with known amounts of surface antibodies. **(B)** Zap70 phosphorylation was detected from lysates of activated Jurkat T cells with low ligand densities (as in Figure 8A) displayed over lipid bilayer-coated glass beads and at different times. **(C)** TIRF images for Jurkat T cells on lipid bilayers with low ligand densities (as in Figure 8A). Cells were stained for pZap70 and CD45 after 3 min of contact with activating ligands on lipid bilayers. **(D)** TIRF images of freshly isolated human T cells on lipid bilayers with low ligand densities (as in Figure 8A). Cells were stained for pZap70 and CD45 after 3 min of contact with activating ligands on lipid bilayers. Ca^2+^ flux measured from Fura-2AM stained Jurkat T cells on a lipid bilayer with low densities (as in Figure 8A) of **(E)** OKT3-1 or **(F)** OKT3-CD43 as well as anti-DNS control scFv (*n* = 25 cells per ligand). Bars, SD.

To examine possible avidity effects associated with high ligand density (Figure S1A in Supplementary Material), we titrated the density of the high-affinity ligands more than 40-folds (Figure 8A). At this lower ligand density, T cells were triggered by both OKT3-1 and OKT3-CD43 as indicated by Zap70 phosphorylation on immunoblots (Figure 8B) and immunofluorescence staining in both Jurkat and primary human T cells (Figures 8C,D). The potency of OKT3-CD43 to induce Zap70 phosphorylation was lower than OKT3-1. Similarly, both high-affinity ligands were able to induce Ca^2+^ mobilization in Jurkat T cells, but with stronger flux induced by OKT3-1 as compared to OKT3-CD43 (Figures 8E,F).

**Figure 9:**
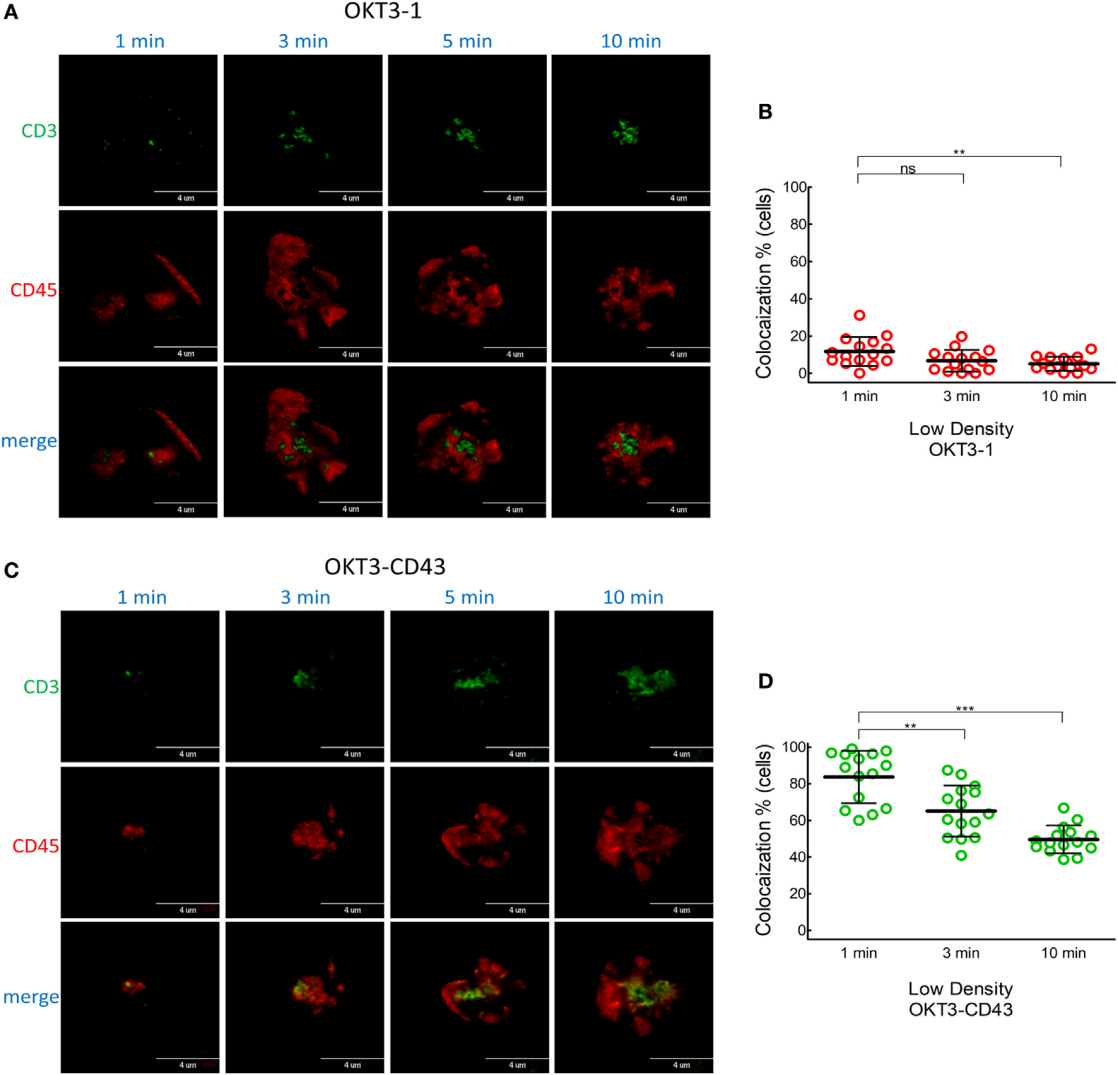
CD45 and CD3 co-localization in T cells activated by low densities of membrane-tethered anti-CD3 scFv. Co-localization of CD3 and CD45 induced by low densities of OKT3-1 and OKT3-CD43 as measured in Figure 8A. **(A)** A representative cell was imaged for CD3 (green) and CD45 (red) by TIRF microscopy at 1, 3, 5, and 10 min after contact with OKT3-1 on a planar lipid bilayer. **(B)** Co-localization percentage between CD3 and CD45 measured by intensity of overlapping pixels for whole Jurkat T cells activated by OKT3-1 (*n* = 15 cells). Bars, SD. **(C)** A representative cell was imaged for CD3 (green) and CD45 (red) by TIRF microscopy at 1, 3, 5, and 10 min after contact with OKT3-CD43 on a planar lipid bilayer. **(D)** Co-localization percentage between CD3 and CD45 measured by intensity of overlapping pixels for whole Jurkat T cells activated by OKT3-CD43 (*n* = 15). Bars, SD.

Low densities of OKT ligands induced patterns of CD45 segregation similar to those induced at high ligand densities; CD45 segregated from CD3 at very early times when cells were triggering with short ligand OKT3-1 (Figures 9A,B), while a high degree of co-localization between CD45 and CD3 was detected for the elongated high-affinity ligand OKT3-CD43 (Figure 9C). In common with high density ligands, segregation of CD3 and CD45 progressively increased at 3 and 10 min when T cells were triggered with low densities of OKT3-CD43 (Figure 9D). This suggests that CD45 segregation is a downstream result of successful triggering at both high- or low-ligand densities.

### CD45 Slowly Segregates in T Cells Incubated With Elongated Low-Affinity Anti-CD3 scFv

Since there was no significant segregation of CD45 from TCR microclusters on T cells incubated for 10 min on lipid bilayers decorated with the low-affinity elongated OKT3^MA^-CD43 scFv, we examined if segregation might occur on longer time scales. Indeed, CD45 partially segregated from TCR microclusters after 1 h of contact between Jurkat T cells and OKT3^MA^-CD43 scFv on lipid bilayers (Figures S3A,B in Supplementary Material).

## Discussion

It is commonly believed that segregation of the membrane tyrosine phosphatase CD45 from engaged TCRs can initiate T cell signaling by creating an environment favorable for phosphorylation of CD3 ITAMs. In the present study, we used lipid membrane-tethered anti-CD3 antibodies to further investigate the dependence of TCR triggering on CD45 segregation. An elongated high-affinity anti-CD3 scFv effectively induced T cell proliferation and cytokine secretion as well as rapidly induced calcium mobilization in human T cells even though it poorly segregated CD45 from TCR microclusters. On the other hand, a low-affinity anti-CD3 scFv with identical dimensions also produced similar poor segregation of CD45 at early times but did not activate T cells. Our results suggest that segregation of CD45 from engaged TCR microclusters may not be absolutely required for initial triggering of TCR signaling.

It has been difficult to definitively show that CD45 segregation is required for TCR triggering because membrane-tethered pMHC molecules with long tethers cannot activate T cells (16, 17, 21). The limitation of using small TCR ligands means that one cannot easily differentiate whether physical segregation of large CD45 molecules from smaller engaged TCR/pMHC complexes is a natural consequence of passive sorting processes that tend to segregate intercellular receptor complexes with different sizes (36) or is a key step initiating TCR triggering (12). Furthermore, the lack of T cell activation by elongated pMHC molecules is consistent with both the KS model (37, 38) and mechanical force models of TCR triggering (39). We recently reported that high-affinity TCR ligands with long tethers can effectively trigger TCR signaling and T cell activation (21), suggesting that segregation of CD45 may not be mandatory for initial triggering of TCR signaling. The OKT3 anti-CD3 antibodies used in our study offers a unique opportunity to examine the role of CD45 segregation on TCR triggering since, in contrast to low-affinity TCR ligands, they effectively activate T cells when anchored to cells *via* both short and elongated tethers (21). Thus, OKT3 scFv anchored on glass-supported lipid bilayers *via* a short one Ig-like domain or an elongated tether derived from the extracellular domain of CD43 induced T cell proliferation and cytokine secretion, rapidly induced phosphorylation of Zap70 and mobilization of intracellular calcium in human T cells. By contrast, a low-affinity scFv variant of the OKT3 scFv (OKT3^MA^), which was generated by introducing two amino acid substitutions in the heavy chain variable region to reduce binding affinity to CD3 by about 250-fold (21), only activated T cells when anchored to membranes via the short one Ig-like domain tether. Thus, in common with low-affinity pMHC ligands (16, 17), both short and elongated membrane-tethered OKT3^MA^ could bind human T cells to a similar degree, but only OKT3^MA^-1 could activate T cells.

**Figure 10:**
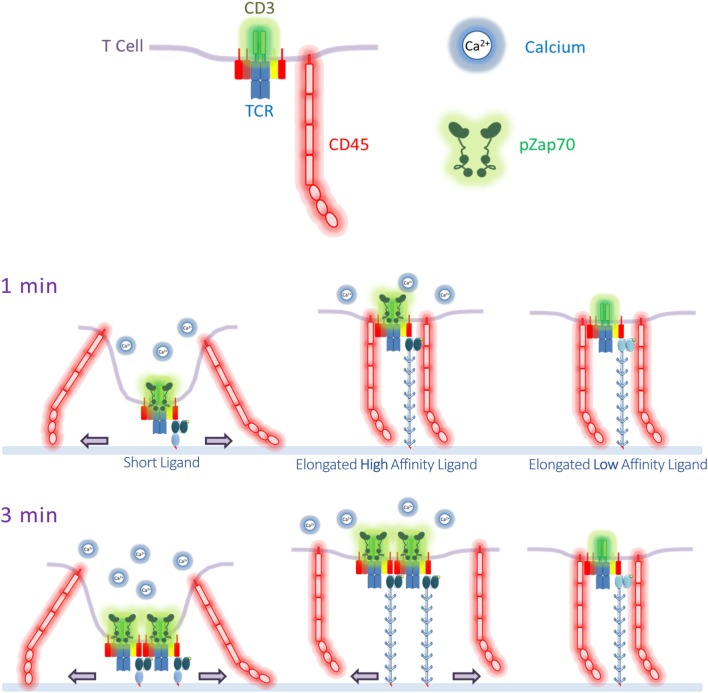
Summary of T cell receptor (TCR) triggering by anti-CD3 scFv ligands. After 1 min of T cell contact to the lipid bilayers decorated with the ligands, short ligands efficiently and completely segregate CD45 from TCRs, accompanied with calcium mobilization. On the other hand, CD45 is co-localized with TCR in cells triggered with elongated ligands, but only a high-affinity elongated ligand was able to induce calcium mobilization to similar levels as the short ligand. However, an elongated low-affinity ligand did not show any segregation of CD45 from TCR, and failed to induce calcium flux. After 3 min, CD45 started to segregate partially from TCR microclusters engaged by an elongated high-affinity ligand but not the low-affinity ligand. pZap70 was also detected in T cells incubated on lipid bilayers decorated with short ligands and elongated high-affinity ligands.

TIRF imaging analysis showed that CD45 mostly segregated from TCR microclusters within 1 min of contact with OKT3-1 or OKT3^MA^-1 as indicated by low co-localization percentages of 4 and 3.6%, respectively. By contrast, CD45 mostly co-localized with TCR microclusters after contact with OKT3-CD43 or OKT3^MA^-CD43 for 1 min (81.3 and 83.3%, respectively) (Figure 5). These differences in CD45 co-localization are consistent with enhanced exclusion of CD45 at the closer contact sites afforded by the short tether (OKT3-1 and OKT^MA^-1) as compared to the longer tether (OKT3-CD43 and OKT3^MA^-CD43). However, even though OKT3-CD43 poorly segregated CD45, it activated T cells equally as well as OKT3-1 as measured by T cell proliferation and cytokine secretion. OKT3-CD43 also induced strong calcium mobilization in T cells within 1 min when there was no significant difference in CD45 segregation between OKT3-CD43 and OKT3^MA^-CD43, which did not induce calcium mobilization, cytokine secretion or proliferation of T cells. Taken together, these results indicate that the degree of CD45 segregation from TCR microclusters does not correlate with initiation of TCR triggering (Figure 10).

The present results are consistent with our recently published data in which we reported that elongated high-affinity ligands can effectively activate T cells, possibly due to forces generated by thermally induced stochastic membrane displacements (21). In particular, we observed that membrane-tethered OKT3 scFv induced strong TCR triggering even when elongated by very long tethers (21). A range of affinities has been reported for OKT3. In one study, OKT3 displayed very low affinity (*K_D_* = 2.63 µM) (40). However, this study used surface plasmon resonance to measure OKT3 Fab binding to recombinant CD3, which may not display the same structure as native CD3 on T cells. By contrast, other studies have measured much higher affinities (*K_D_* = 5 × 10^−10^ M) for OKT3 (41, 42). We also previously observed that soluble OKT3 scFv displayed the highest affinity among several anti-CD3 antibodies examined with an apparent *K_D_* in the low nanomolar range (21). By contrast, OKT3^MA^ displayed about 250-fold lower binding affinity as compared to OKT3 (21). Of note, the OKT3 affinity is very high as compared to the affinity of physiological ligands (pMHC), which is in the micromolar range (43, 44).

The high ligand densities used in our study may increase the proximity of TCR microclusters, driving cross-linking, and isolating TCR islands from CD45. To examine this possibility, we titrated ligand densities by more than 40-folds. Our results show that at the lower ligand densities, TCRs can still be triggered by high-affinity ligands as measured by Zap70 phosphorylation and Ca^2+^ mobilization. This is in agreement with previously published data using the same ligands tethered to the surface of 3T3 fibroblasts, where both OKT3-1 and OKT3-CD43 were able to induce cytokine secretion in Jurkat T cells at low densities (21). Clustering of TCRs seems unlikely to be the sole cause of triggering by TCR ligands because TCRs are already clustered on naïve inactivated T cells (45, 46), and mostly concentrated at T cell protrusions or microvilli (47) where contact to APCs often begins (48) as compared to lower TCR density at the T cell body (47). In addition, although Lck can be found free or bound to the T cell coreceptor, initial TCR triggering is induced by free Lck (49). Moreover, many soluble anti-CD3 or anti-TCR antibodies, which should cluster TCRs, cannot activate T cells unless they are first immobilized on accessory cells or a plastic surface or beads (50, 51). Soluble pMHC dimers linker *via* a short spacer can induce strong T cell activation whereas soluble pMHC dimers linker *via* long spacers cannot activate T cells (52), even though they can both cluster TCRs. This result, is more consistent with the “permissive geometry” model in which the TCR orientation in the membrane is altered by the smaller pMHC dimers (45). Finally, T cells can be activated by less than five and possibly even a single pMHC molecules where clustering should be minimal (53, 54). Taken together, these studies argue that TCR clustering may not be the mechanism responsible for TCR triggering.

Several studies examined the effect on T cell activation of truncating or altering the large ectodomain of CD45 or other membrane tyrosine phosphatases, which may increase their co-localization with engaged TCRs. Indeed, Irles and colleagues showed that TCR triggering is reduced in cells expressing a truncated form of CD45 (55). However, soluble antibodies which cannot physically exclude CD45 from TCRs could activate T cells expressing full length CD45, while poor T cell signaling was observed in cells expressing truncated CD45 (55). Lin and Weiss showed that shortening the extracellular domain of CD148 disrupts its exclusion from the immune synapse and greatly diminished NFAT activation and translocation to the nucleus (56). However, exclusion of CD148 from the immune synapse may primarily act to prolong TCR signaling rather than control TCR triggering. In another study, replacement of the CD45 external domain with the ectodomain of EGFR did not affect T cell activation as compared to T cells that expressed wild type CD45ABC or CD45RO (57). Similarly, expression in CD45-deficient Jurkat T cells of a CD45 chimera in which the transmembrane and extracellular domains of CD45 were replaced with the corresponding domains from MHC-I (HLA-A2) was shown to rescue proximal TCR signaling (58). Likewise, expression of a chimeric molecule in which a myristoylation domain was fused to the intracellular portion of CD45 to create a membrane-associated form of CD45 with no extracellular domain totally restored tyrosine phosphorylation and calcium mobilization in CD45-deficient T cells (59). In an elegant cell reconstitution system, chimeric proteins in which the large CD45 ectodomain was swapped with smaller CD2 or CD86 ectodomains resulted in increased localization of chimeric CD45 molecules in the T cell-APC interface and prevented the recruitment of Zap70 to the interface, indicating TCR triggering was abolished (60). However, both CD2 and CD86 can bind receptors on APCs, which may alter the activity of CD45 as previously demonstrated for other chimeric CD45 receptors (57). Forcing T cells to spread in close contact to substrate can also physically drive CD45 and TCR segregation, and allow TCRs to be non-specifically triggered (14). However, membrane bending induced at sites of close cell contact to accommodate larger membrane proteins might also trigger nearby TCRs by providing new docking or recognition sites [reviewed in Ref. (61, 62)], altering association of CD3 cytoplasmic tails with the inner plasma membrane (63–65) or by activating the membrane-associated mechanosensor protein Piezo1, which was recently found to regulate mechanosensing in T cells (66). Rather than acting as the initial trigger for TCR activation, CD45 segregation may play an important role to prolong signaling from engaged TCRs by preventing dephosphorylation of CD3 and pZap70 (37, 38). A recent study using reconstituted proteins demonstrated that the negatively charged CD45 cytoplasmic domains are excluded from assembled microclusters of phosphorylated LAT, thereby prolonging their phosphorylation (67). This mechanism seems to be independent of the extracellular domain of CD45. This may explain why poor segregation of CD45 at the initial time of triggering by the elongated high-affinity ligand did not affect signaling.

We suggest that CD45 segregation from engaged TCRs may occur by three mechanisms: i. size-dependent sorting of membrane proteins, ii. microcluster-based phase separation, and iii. cytoskeleton-driven sorting of engaged TCRs. Surface proteins naturally rearrange into zones with similar extracellular sizes at interfacial contact sites to minimize energy associated with membrane bending (36, 68). Proteins with larger size discrepancies should sort faster than proteins with more similar dimensions. Thus, CD45 [which ranges in size from ~ 22 to 55 nm depending on the specific isoform (14)] segregated from OKT3-1 and OKT3^MA^-1 anti-CD3 scFv contact sites (~10 nm) (Figure S4A in Supplementary Material) immediately upon ligation, but substantially co-localized with OKT3-CD43 or OKT3^MA^-CD43 (average of 19 nm but with wide range of variation up to 40 nm) at a minute of contact (Figure S4B in Supplementary Material). This result is consistent with relatively little driving force to physically segregate CD45 from the large TCR ligands. It is also important to mention that bigger clusters showed a high degree of segregation, probably because they grow up with time, accumulating more TCRs and segregating CD45 by size distribution on the cell interface, which favors the lowest degree of membrane bending (the lowest free energy) (Figure S5 in Supplementary Material). We also observed that TCR microclusters formed with OKT3-CD43 segregated from CD45 in comparison to TCR microclusters formed with the non-activating OKT3^MA^-CD43 scFv. Upon TCR activation, the phosphorylated CD3ζ ITAMs provide docking site for the protein kinase Zap70. The substrates of pZap70 protein kinase are LAT, SLP76 and PLC-γ1 (69, 70). The phosphorylated scaffold protein pLAT and the associated phosphorylated SLP76 are sites for recruiting many other proteins, such as VAV, GADS, and NCK (71). The formed pLAT signalosome can exclude negatively charged proteins, such as CD45, which has a cytoplasmic tail with an isoelectric point of 6.4 (67), suggesting that CD45 segregation follows TCR triggering and microcluster formation. Activated TCRs are connected to the actin cytoskeleton under the regulation of myosin IIA that drives movement of TCRs toward the center of the immunological synapse (72–75). Such movement requires TCR triggering, which is provided by short ligands, as well as OKT3-CD43 but not OKT3^MA^-CD43. The differential segregation of TCR microclusters formed with elongated anti-CD3 scFv may be explained by OKT3-CD43 initiated cytoskeleton-driven movement of TCR microclusters away from CD45.

Several studies have reported that the TCR can act as a mechanosensor (18, 20, 76–80). Newly activated TCR microclusters are formed in the pSMAC which contains a high density of proteins with large ectodomains, such as CD45, CD43, CD148, and ICAM-1/LFA-1 complexes (81). In particular, TCR triggering by physiological ligands depends on the formation of LFA-1/ICAM-1 micro-adhesion rings that surround newly generated TCR microclusters (82). Under physiological conditions, localized membrane bending on the nanometer scale may be required to accommodate smaller TCR/pMHC complexes among the sea of larger receptors, thereby generating sufficient forces to induce TCR triggering. Constraining TCRs in close contact to an opposing surface *via* CD2–CD48 interactions was recently shown to induce non-specific TCR triggering (14). This result is explained by the KS model as segregation of CD45 from TCRs at sites of close cell contacts. However, enforced close contact of opposing cell surfaces may also generate membrane bending to accommodate larger membrane proteins, which might also drive TCR triggering. Recently, Liu and colleagues showed that T cell activation is regulated by the membrane-associated mechanosensor Piezo1, suggesting that mechanical pressure applied to the T cell membrane may be involved in TCR triggering (66).

Mechanical force models predict that elongation of membrane-tethered ligands will reduce tensive forces as the size differential between engaged TCRs and surrounding cell surface receptors decreases. Indeed, T cell activation is progressively lost as membrane-tethered low-affinity ligands are elongated by 10–30 nm (16, 17, 21). However, we observed in this and a previous study (21) that elongation of high-affinity ligands does not abrogate their ability to activate T cells. A possible explanation for this result is that high-affinity ligands such as OKT3-CD43 bind to TCRs for sufficient times to generate forces by additional mechanisms besides membrane bending. Thermal energy at physiological temperatures causes cell membranes to rapidly fluctuate with submicron displacements at frequencies of 0.1–1 s (83–86). Stochastic membrane fluctuations may allow accumulation of triggering forces on TCRs that are engaged by high-affinity ligands for sufficient times (87). Recently, Pullen and Abel showed by mathematical modeling that strong agonists generate long-lived catch bonds that can withstand forces generated by fluctuating membranes (87). OKT3-CD43, but not the lower affinity OKT3^MA^-CD43, may, therefore, generate catch bonds that accumulate sufficient forces to drive T cell activation (20).

In summary, we demonstrate that TCRs can be activated by elongated high-affinity ligands without apparent segregation of CD45. We suggest that CD45 can segregate from engaged TCRs due to phase separation from TCR microclusters, physical sorting into regions containing similarly sized receptors, and cytoskeleton-mediated sorting of activate TCR clusters, but that these mechanisms act to prolong TCR signaling rather than initiate triggering of TCRs.

## Ethics Statement

All animal experiments were carried out in accordance with the recommendations of Institutional Animal Care and Utilization Committee (IACUC) guidelines at Academia Sinica. The protocol was approved by the IACUC and Laboratory Animal Facility and Pathology Core Committee of the Institute of Biomedical Sciences, Academia Sinica. Human whole blood, obtained from healthy donors by the Taipei City Blood Bank, was used under procedures approved by the Academia Sinica Human Subject Research Ethics Committee (protocol AS-IRB01-11069).

## Author Contributions

MA-A and SR designed experiments and wrote the manuscript, MA-A performed experiments, and Y-SC and B-MC provided technical help.

## Conflict of Interest Statement

The authors declare that the research was conducted in the absence of any commercial or financial relationships that could be construed as a potential conflict of interest.
