# Supplementary material for: High-Affinity Ligands Can Trigger T Cell Receptor Signaling Without CD45 Segregation
Source: Front Immunol. 2018 Apr 9;9:713. doi: 10.3389/fimmu.2018.00713 (PMC5900011; doi:10.3389/fimmu.2018.00713)
Supplement: Supplementary file 3 [file data_sheet_1.PDF]

frontiers

High affinity ligands can trigger T cell receptor signaling without CD45 segregation

Mohammad Ameen Al-Aghbar, Yeh-Shiu Chu, Bing-Mae Chen, Steve R Roffler

Supplementary Materials

Supplementary Figures

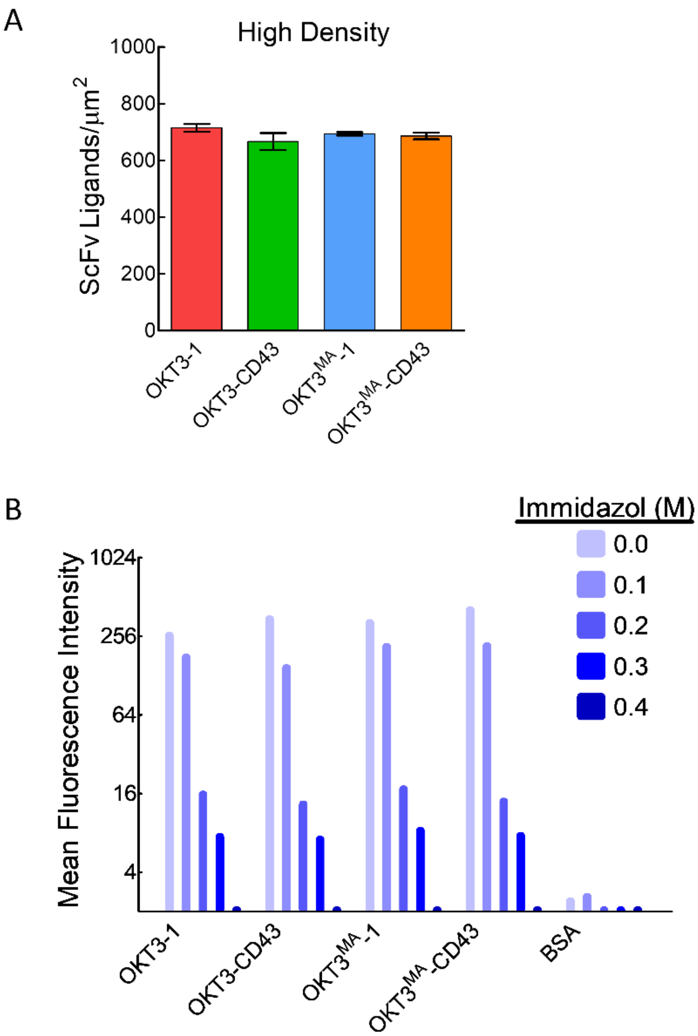

Supplementary Figure 1. **Binding of anti-CD3 scFv to glass-supported lipid-bilayers. A.** Estimated densities of anti-CD3 scFv on lipid bilayers measured by comparing fluorescent intensities of anti-CD3 scFv on lipid bilayer coated glass beads relative to standard beads with known coated surface antibodies. **B.** Mean fluorescence intensities of stained lipid bilayer-coated glass beads decorated by scFv ligands. Staining was performed by 1 µg/ml 2% FBS in PBS rat anti-HA followed by 1 µg/ml 2% FBS in PBS goat anti-rat IgG-FITC at room temperature for 40 minutes each. Different concentrations of imidazole were added to strip the ligands from the lipid bilayer-coated beads, the mean fluorescence intensities were measured by flow cytometry.

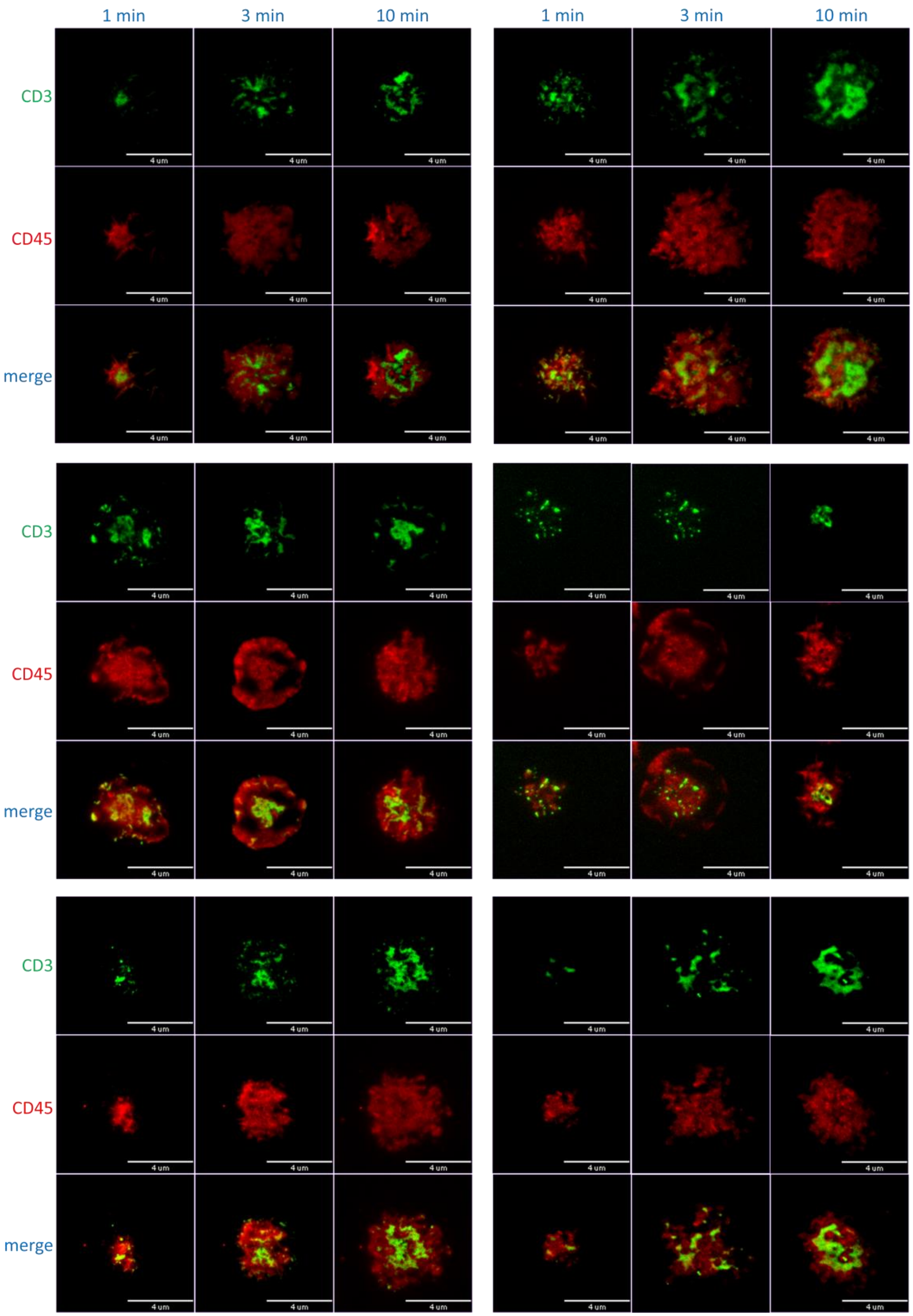

Supplementary Figure 2. Partial CD45 segregation from CD3 by T cells activated by OKT3-CD43. Representative 6 Jurkat T cells were imaged for CD3 (green) and CD45 (red) by TIRF microscopy at 1, 3, and 10 min after contact with OKT3-CD43 on a planar lipid bilayer**.**

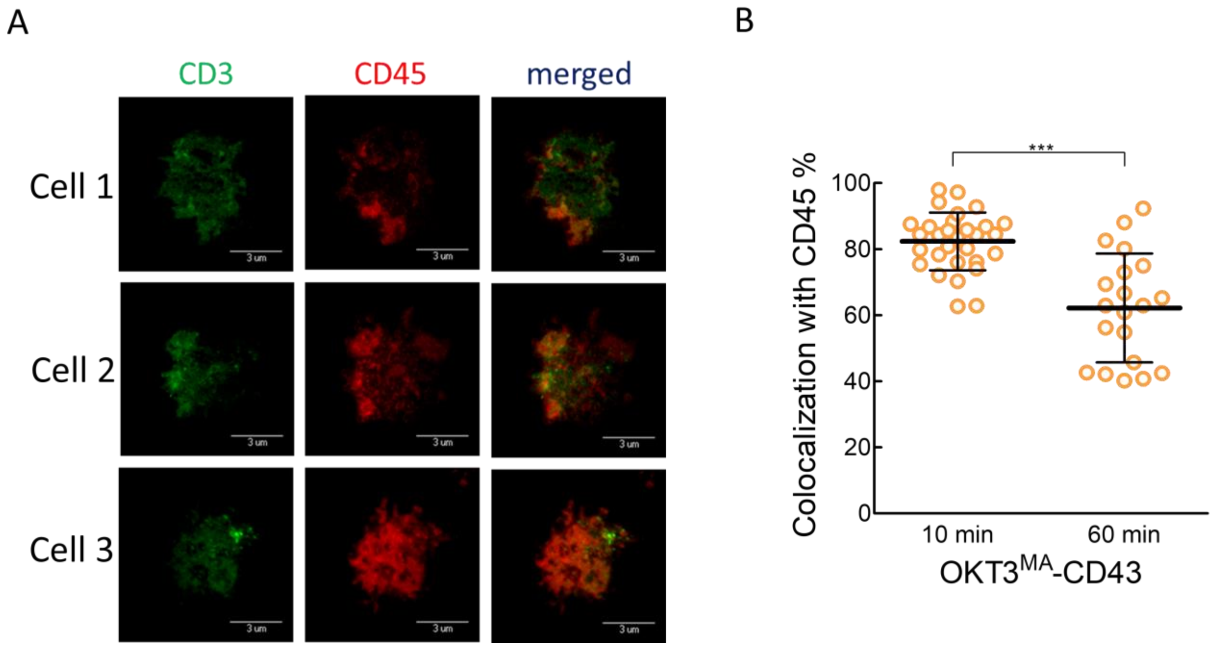

Supplementary Figure 3. Non-activated T cells incubated on bilayers coated with OKT3^{MA}-CD43 slowly rearrange proteins based on dimensions. Jurkat T cells were dropped over planar lipid bilayers decorated with the elongated low affinity ligand OKT3^{MA}-CD43. A. TIRF microscopy images for CD3 (green) and CD45 (red) were taken after an hour of adding cells. The figure shows 3 representative cells. B. Colocalization percentage between CD3 and CD45 measured by intensity of overlapped pixels for whole Jurkat cell activated by OKT3^{MA}-CD43 for 10 and 60 min (n= 30 and 20 cells, respectively). Bars, SD.

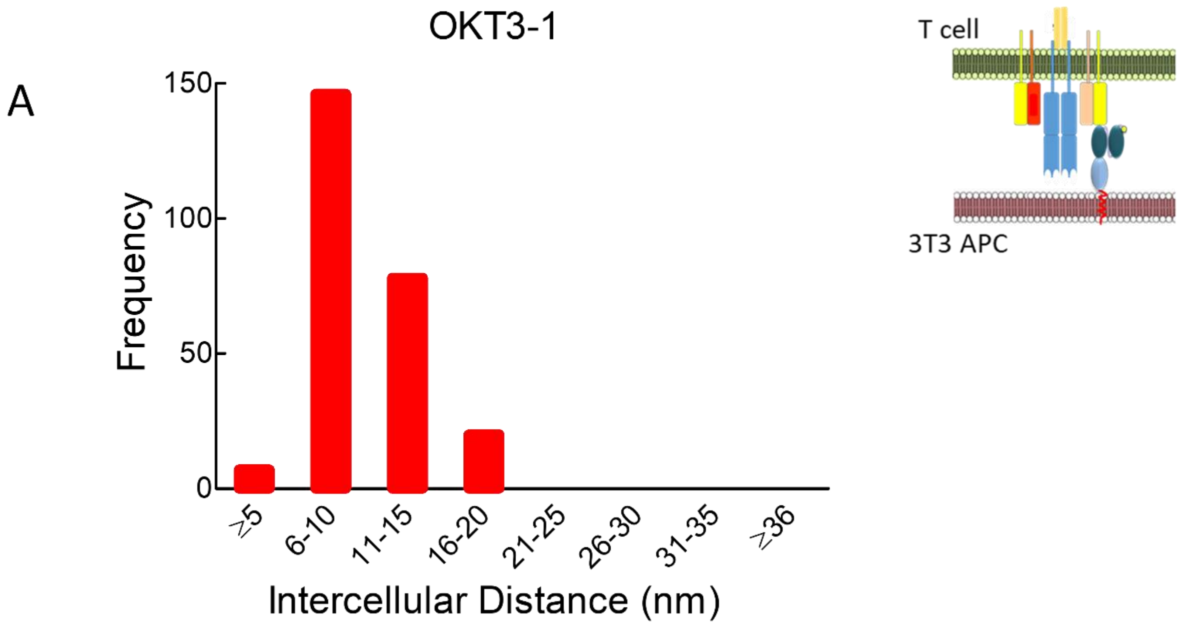

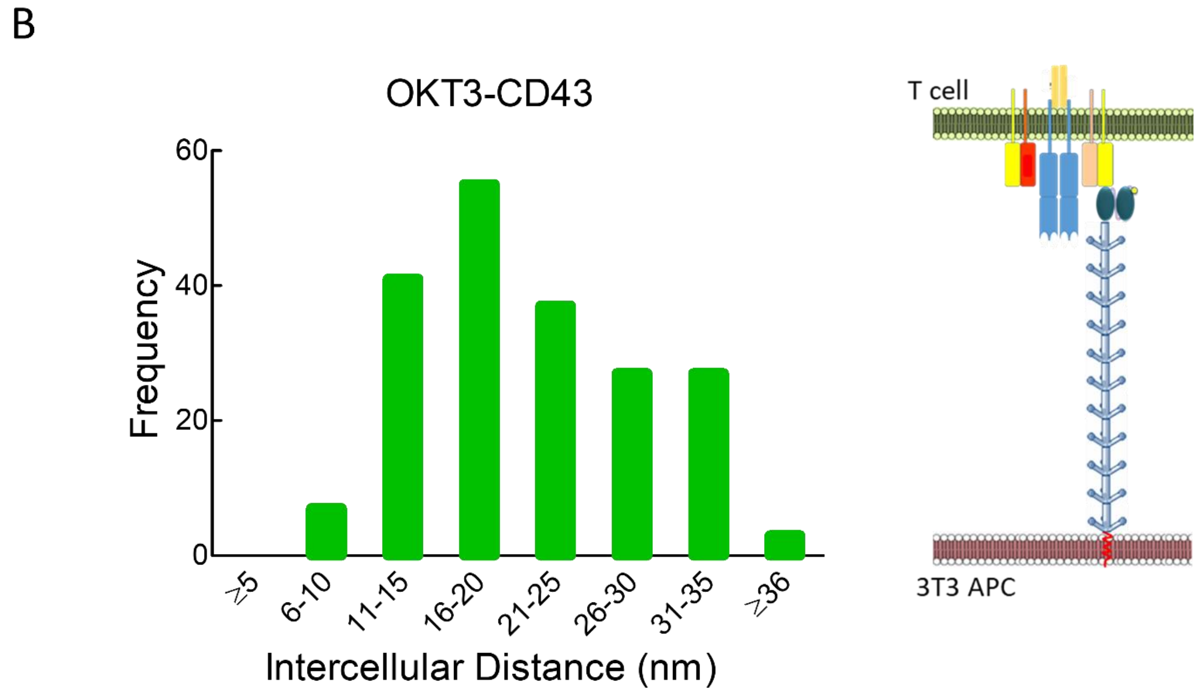

Supplementary Figure 4. Intercellular distance between 3T3 APC expressing short and long OKT3 and Jurkat T cells. Jurkat T cells were incubated with cultured 3T3 cells monolayer expressing membrane-tethered OKT3-1 (A) or OKT3-CD43 (B) for 2 hours before being fixed and processed for EM microscopy as described by Chen et al. 2017 (21). Using Adobe Photoshop CS5, equal spaced sections were obtained from the conjugation area between two cells, and the distance of each point was measured. The frequency of each length incident was indicated in the figure.

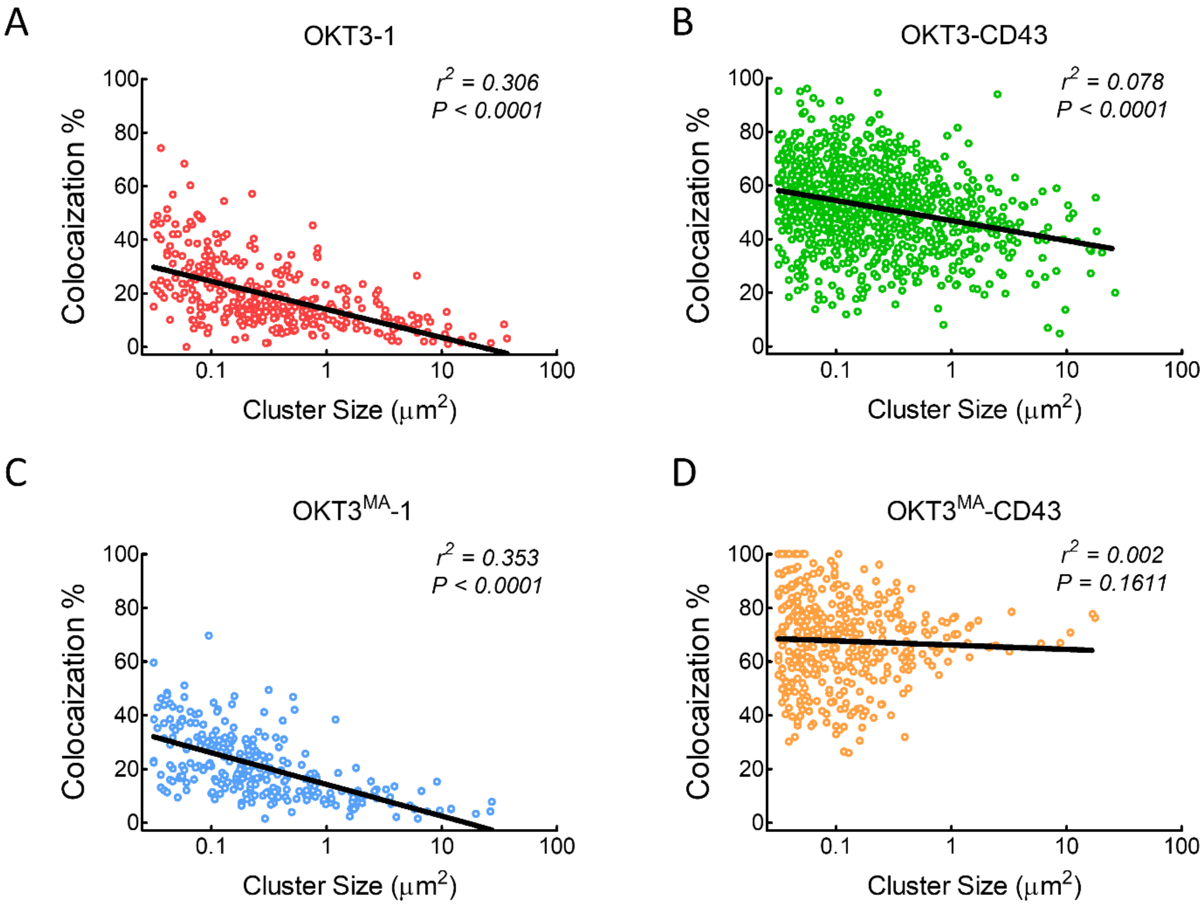

Supplementary Figure 5. Bigger CD3 microclusters efficiently segregate CD45. We plotted the data for the cluster sizes in figures 4C, 4F, 5C, and 5F for the 3 times (1, 3, and 10 min) versus the measured colocalization, and then performed linear regression analysis. **(A)** OKT3-1. **(B)** OKT3-CD43. **(C)** OKT3^{MA}-1. **(D)** OKT3^{MA}-CD43.

Supplementary Videos

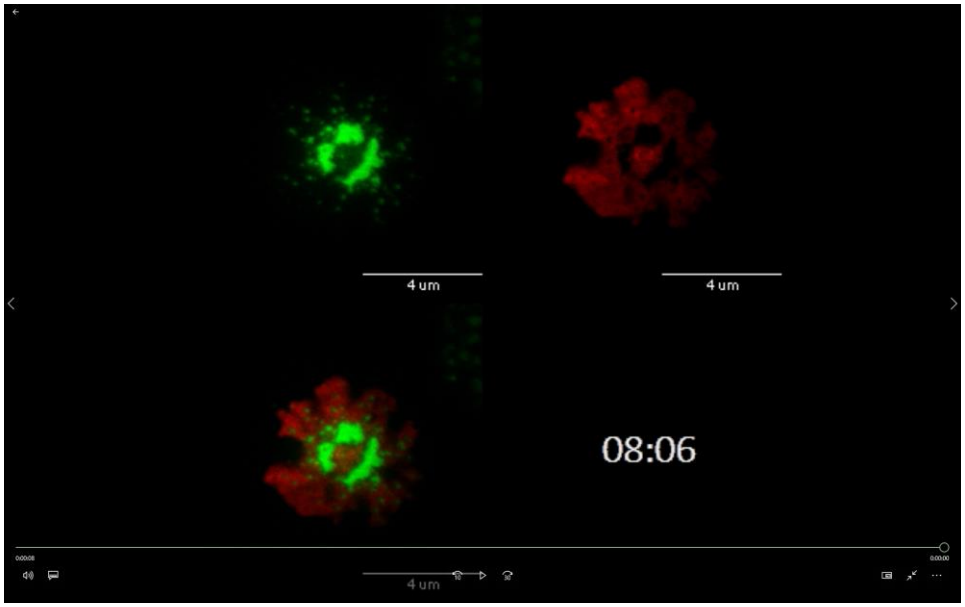

Supplementary video 1. Triggering T cell with OKT3-1. Live TIRF imaging of representative Jurkat T cell upon contacting a lipid bilayer coated glass surface grafted with OKT3-1. Green represents CD3 while red represents CD45. Time is shown by minutes and seconds.

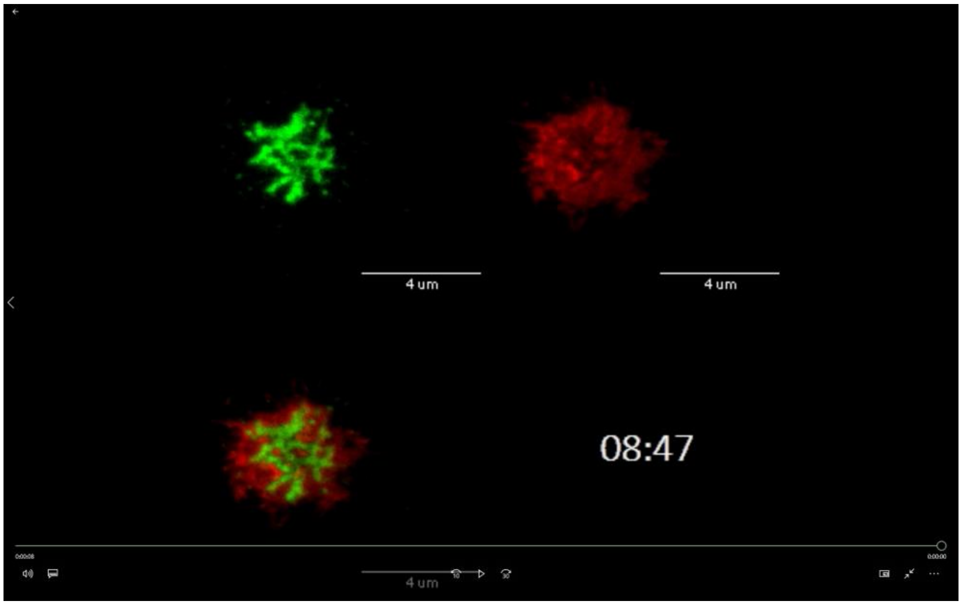

Supplementary video 2. Triggering T cell with OKT3-CD43. Live TIRF imaging of representative Jurkat T cell upon contacting a lipid bilayer coated glass surface grafted with OKT3-CD43. Green represents CD3 and while represents CD45. Time is shown by minutes and seconds.
